# AP4 suppresses DNA damage, chromosomal instability and senescence via inducing *MDC1/Mediator of DNA damage Checkpoint 1* and repressing *MIR22HG/*miR-22-3p

**DOI:** 10.1186/s12943-022-01581-1

**Published:** 2022-05-27

**Authors:** Jinjiang Chou, Markus Kaller, Stephanie Jaeckel, Matjaz Rokavec, Heiko Hermeking

**Affiliations:** 1 Experimental and Molecular Pathology, Institute of Pathology, Ludwig-Maximilians-University, Thalkirchner Strasse 36, 80337 Munich, Germany; 2 German Cancer Consortium (DKTK), Partner site Munich, Munich, Germany; 3 German Cancer Research Center (DKFZ), Heidelberg, Germany

**Keywords:** *AP4*, *c-MYC*, *MIR22HG*, miR-22-3p, *MDC1*, DNA damage, DNA repair, Homologous recombination, Chemo-resistance, Colorectal cancer

## Abstract

**Background:**

*AP4 (TFAP4)* encodes a basic helix-loop-helix leucine zipper (bHLH-LZ) transcription factor and is a direct target gene of the oncogenic transcription factor c-MYC. Here, we set out to determine the relevance of AP4 in human colorectal cancer (CRC) cells.

**Methods:**

A CRISPR/Cas9 approach was employed to generate *AP4*-deficient CRC cell lines with inducible expression of c-MYC. Colony formation, β-gal staining, immunofluorescence, comet and homologous recombination (HR) assays and RNA-Seq analysis were used to determine the effects of AP4 inactivation. qPCR and qChIP analyses was performed to validate differentially expressed AP4 targets. Expression data from CRC cohorts was subjected to bioinformatics analyses. Immunohistochemistry was used to evaluate AP4 targets in vivo. *Ap4*-deficient *APC*^min/+^ mice were analyzed to determine conservation. Immunofluorescence, chromosome and micronuclei enumeration, MTT and colony formation assays were used to determine the effects of AP4 inactivation and target gene regulation on chromosomal instability (CIN) and drug sensitivity.

**Results:**

Inactivation of *AP4* in CRC cell lines resulted in increased spontaneous and c-MYC-induced DNA damage, chromosomal instability (CIN) and cellular senescence. *AP4*-deficient cells displayed increased expression of the long non-coding RNA *MIR22HG,* which encodes miR-22-3p and was directly repressed by AP4. Furthermore, *Mediator of DNA damage Checkpoint 1* (*MDC1*), a central component of the DNA damage response and a known target of miR-22-3p, displayed decreased expression in *AP4*-deficient cells. Accordingly, *MDC1* was directly induced by AP4 and indirectly by AP4-mediated repression of miR-22-3p. Adenomas and organoids from *Ap4*-deficient *APC*^min/+^ mice displayed conservation of these regulations. Inhibition of miR-22-3p or ectopic *MDC1* expression reversed the increased senescence, DNA damage, CIN and defective HR observed in *AP4*-deficient CRC cells. *AP4*-deficiency also sensitized CRC cells to 5-FU treatment, whereas ectopic AP4 conferred resistance to 5-FU in a miR-22-3p and MDC1-dependent manner.

**Conclusions:**

In summary, AP4, miR-22-3p and *MDC1* form a conserved and coherent, regulatory feed-forward loop to promote DNA repair, which suppresses DNA damage, senescence and CIN, and contributes to 5-FU resistance. These findings explain how elevated AP4 expression contributes to development and chemo-resistance of colorectal cancer after c-MYC activation.

**Graphical abstract:**

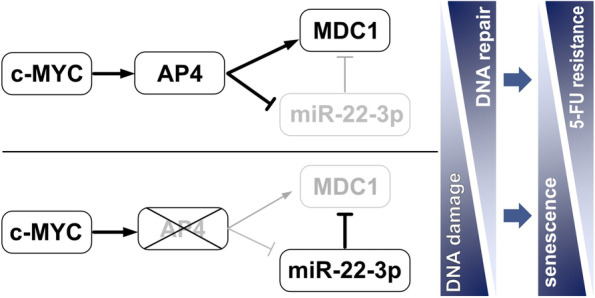

**Supplementary Information:**

The online version contains supplementary material available at 10.1186/s12943-022-01581-1.

## Background

Colorectal cancer (CRC) is one of the most common human malignancies accounting for approximately 10% of worldwide cancer incidence and mortality [1]. While early-stage CRC is curable by surgery, treatment of metastatic colorectal cancer (mCRC) remains an unmet clinical need. Moreover, about 25% of CRC cases are diagnosed only at the metastatic stage. Despite the extensive molecular and functional knowledge on this disease, systemic therapy for mCRC still relies on traditional 5-fluorouracil (5-FU)-based chemotherapy regimens [2]. So far, targeted therapies and immunotherapy have shown effectiveness only in a limited subset of CRC patients. Therefore, there is an urgent need to understand the molecular mechanisms and regulations underlying CRC development and treatment resistance in order to implement novel, rationally driven, tailored therapies.

AP4 (TFAP4) is a basic helix-loop-helix leucine zipper (bHLH-LZ) transcription factor that exclusively forms homodimers, which bind to the E-box motif CAGCTG [3]. We previously identified the *AP4* gene as a direct transcriptional target of c-MYC [4]. In the intestine, AP4 expression is confined to progenitor and stem cells [4–6]. Deletion of AP4 causes premature senescence and defects in mitogen-induced proliferation in mouse embryonic fibroblasts (MEFs) [5, 7]. Furthermore, AP4 expression is strongly elevated in several types of cancer [5, 8, 9]. AP4 presumably contributes to the phenotype of cancer cells by activating or repressing genes that harbor CAGCTG elements in their promoter regions, thereby controlling processes such as proliferation, metabolism, apoptosis, epithelial-mesenchymal transition (EMT) and metastasis [5]. In addition, AP4 was shown to maintain a c-MYC-induced transcriptional program in murine T cells after exposure to IL2 once c-MYC is down-regulated [10]. Recently, we showed that *Ap4* is critical for adenoma initiation and growth by controlling the homeostasis of intestinal stem cells in the *APC*^min/+^ mouse model of intestinal cancer [6]. However, the molecular mechanisms by which the c-MYC/AP4 axis promotes CRC development and progression are still largely unknown.

In the current study, we show that *AP4* deletion in CRC cells results in increased DNA damage, senescence, chromosomal instability and a decrease in homologous recombination (HR). Comparative analysis of transcriptional profiles obtained from *AP4*-deficient CRC cells implicated *MIR22HG* and *MDC1/Mediator of DNA Damage Checkpoint* as AP4 target gene candidates that may be relevant for the increase in DNA damage observed after *AP4* deletion. Indeed, AP4 directly activated *MDC1* expression and repressed miR-22-3p, which targets MDC1, thereby further enhancing *MDC1* expression. In addition, we could show that the AP4/miR-22-3p/MDC1 axis is important for an effective response to spontaneous and c-MYC-induced DNA damage by increasing DNA repair by HR. Therefore, deregulation of the AP4/miR-22-3p/MDC1 axis by an activated Wnt/c-MYC pathway, which is a hallmark of CRCs, may ultimately contribute to chemo-resistance.

## Materials and methods

### Cell culture and treatments

The CRC cell lines DLD-1 and SW480 were cultured in McCoy’ 5A medium (Invitrogen, Carlsbad, CA, USA) with 10% fetal bovine serum (FBS) (Invitrogen) containing 100 units/ml penicillin and 0.1 mg/ml streptomycin in 20% O_2_, 5% CO_2_ and 37 °C. Doxycyclin (DOX) was purchased from Sigma-Aldrich (St. Louis, MO, USA) and dissolved in water (100 μg/ml stock solution). The final concentration of DOX used in cell treatment was 100 ng/ml. To maintain cell pools harboring pRTR vectors, a final concentration of 8 μg/ml puromycin was used and changed fresh medium every 2 days. pRTR vectors and pcDNA3.1 vectors were transfected with Lipofectamine 2000 (Invitrogen). MiRNA mimic, siRNAs and negative controls were transfected with Lipofectamine RNAiMAX Transfection Reagent (Invitrogen). For transfections the final concentration was 500 ng/ml for plasmids and 12.5 nM for RNAs. miR-22-3p mimics, antagomirs and controls were purchased from Qiagen (Hilden, Germany). The sequence information of miR-22-3p mimic and controls are listed in Table S1.

### Generation of *AP4*-deficient cell lines

To generate single cell clones with complete AP4 protein expression abrogation, a CRISPR/Cas9 approach was employed. We designed three guide RNAs (listed in Table S2) targeting exon 2 of the *TFAP4/AP4* gene, and cloned each of them as two complementary DNA oligonucleotides into the BbsI sites of pSp-Cas9-GFP to generate single-guide (sg) RNA expression plasmids, as described previously [11]. DLD-1 and SW480 cells were then transfected with 2 μg of each pSp-Cas9-sgRNA-GFP plasmid, or transfected with “empty” pSp-Cas9-GFP harboring no guide RNA sequence. Forty-eight hours posttransfection, GFP-positive cells were sorted into 96-well plates using a FACSARIA cell sorter (BD Biosystems, NJ, USA) and expanded as single-cell clones for 2 weeks before screening by Western blot analysis. Cells transfected with pSp-Cas9-GFP harboring no guideRNA sequence were treated in a similar manner to obtain *AP4* wild type single-cell clones.

### Generation of pRTR-c-*MYC*-VSV pools

The DLD-1 *AP4* WT1/KO2 and SW480 *AP4* WT1/KO1 clones were subsequently used for the generation of pRTR-c-*MYC*-VSV pools as described previously [5]. The pRTR vector system allows the stringent control of *c-MYC* expression by addition of DOX to the media. Cell pools with more than 80% of the cells expressing a fluorescent marker protein from the pRTR vector were generated by selection for 2 weeks. The percentage of GFP-positive cells in pools derived from *AP4* WT and KO clones harboring pRTR-*c*-*MYC*-VSV was determined by flow cytometry (CFlow6; Accuri, Ann Arbor, MI, USA).

### Sample isolation from *APC*^min/+^ mice with deletion of *Ap4*

Generation of *APC*^min/+^ mice with inactivation of *Ap4* in intestinal epithelial cells (IEC), isolation of samples and derivation of organoids from IECs and adenomas from these mice has been described before [6]. Mice were kept in individually ventilated cages with a 12-hour light/dark cycle and ad libitum access to water and standard rodent diet. All animal experimentations and analyses were approved by the Government of Upper Bavaria, Germany (AZ 55.2–1-54-2532-4-2014).

### Beta-galactosidase (β-gal) staining

β-gal staining was performed according to instructions provided in Senescence β-Galactosidase Staining Kit (#9860, Cell Signaling Technology, Massachusetts, USA). Briefly, cells were seeded into 6-well plate at the density of 2 × 10^5^ cells/well. For fixation, cells were washed once with PBS and then fixed for 30 min. Next, fixed cells were stained with β-gal staining solution containing X-gal overnight at 37 °C. Cells were imaged by using a microscope (Axiovert 25, Zeiss, Jena, Germany) with Axiovision software (Version 4.8.0.0, Zeiss).

### Immunofluorescence analysis

For detection by indirect immunofluorescence cells were seeded on a round glass in a 6-well plate at the density of 2 × 10^5^ cells/well. After different treatments, cells were fixed with 4% paraformaldehyde in PBS for 20 min. Then 0.2% Triton X 100 was used to permeabilize cells for 5 min at room temperature. Then cells were blocked in 1% BSA/PBS for 1 h at room temperature. γH2AX and MDC1 were detected using the respective antibodies listed in Table S3. Cellular chromatin was stained by DAPI (Roche, Switzerland). Stained cells were covered with ProLong Gold antifade (Invitrogen). Image acquisition was performed with a confocal microscope (LSM 700, Zeiss) and the ZEN 2009 software (Zeiss). Foci quantification was performed with Image J software. Cells with over 10 foci were considered as positive. The fluorescence intensity was normalized to DAPI. For each condition at least three microscope fields with a total of 150 cells were quantified.

### Comet assay

The comet assay was conducted according to instructions provided in Comet Assay Kit (3-well slides, ab238544, Abcam, USA). After detachment by trypsin cells were suspended in ice-cold PBS at 1 × 10^5^ cells/ml. Suspended cells were combined with pre-heated comet agarose (37 °C) at a 1/10 ratio (v/v) and mixed by pipetting. 70–80 μl cell-agarose mixture was added to comet agarose base layer and incubated at 4 °C for 15 min in the dark. Then the slides with agarose were immersed into 25 ml ice-cold lysis buffer for 60 min at 4 °C in the dark. Lysis buffer was replaced with ice-cold alkaline solution and incubated for 30 min at 4 °C in the dark. Slides were immersed with ice-cold alkaline electrophoresis solution for 5 min and then subjected to electrophoresis in cold alkaline electrophoresis solution (electrophoresis condition: 1 V/cm for 10–15 min). Subsequently, slides were immersed twice with pre-chilled water for 2 min. Finally, slides were immersed into 70% ice-cold Ethanol for 1 min and placed in the dark for drying. Signals were evaluated using a confocal microscope (LSM 700, Zeiss) with a FITC filter. For data analysis and DNA tail moment determination the ImageJ software in conjunction with the OpenComet plugin [12] was used.

### RNA isolation and quantitative real-time polymerase chain reaction (qPCR) analysis

Total RNA of cells was isolated and purified by using High Pure RNA Isolation Kit (Roche) based on the protocol provided by the manufacturer. Total RNA from *Apc*^*Min/+*^ adenomas was isolated using the RNAeasy Kit (QIAGEN). 5 adenomas per mouse were used for each sample. For each sample, 1 μg RNA was used to generate cDNA via using Verso cDNA Synthesis Kit (Thermo Fisher Scientific, Waltham, MA, USA). For quantitative real-time polymerase chain reaction (qPCR), a Fast SYBR Green Master Mix (Applied Biosystems, Foster City, CA) was used to perform the reaction in LightCycler 480 (Roche) system. Gene expression was normalized to *GAPDH* or *β-actin* with the ΔΔCt method [13]. Experiments were performed in triplicates. Sequence information of the primers is provided in Table S4.

### Western blot analysis

Cells were lysed in RIPA lysis buffer (50 mM Tris/HCl, pH 8.0, 250 mM NaCl, 1% NP40, 0.5% [w/v] sodium deoxycholate, 0.1% SDS) containing mini protease inhibitors (Roche) and PhosSTOP Phosphatase Inhibitor Cocktail Tablets (Roche). Cell lysates were sonicated for 5 seconds in each sample and centrifuged at 13,000 rpm for 20 min at 4 °C. Supernatants containing proteins were collected and quantified by Pierce™ BCA Protein Assay Kit (Thermo Fisher Scientific). For each sample, total 40 μg protein was loaded and separated by 10% sodium dodecyl sulfate (SDS) polyacrylamide gel electrophoresis. Immobilon PVDF membranes (Millipore, Burlington, MA, USA) were used for transferring after electrophoresis using standard protocols (Bio-Rad Laboratories, Hercules, CA). ECL (Millipore) system was used and imaged through LI-COR Odyssey FC imaging system (Bad Homburg, Germany). Antibodies are provided in Table S3.

### Immunohistochemical analysis

FFPE tissue was cut into 2 μm sections on a microtome and de-paraffinized. After antigen retrieval slides were incubated with primary antibody (the primary antibodies used are listed in Table S3) over night at 4 °C and washed with Tris-HCL (Tris hydrochloride) buffer (pH 7.5) followed by a secondary antibody. Antibodies were detected with the Vectastain Elite ABC (avidin-biotin complex) kit (Vector) using DAB (3,3′-diaminobenzidine) (Vector Laboratories and Dako) for brown stainings or AEC (3-Amino-9-ethylcarbazole) (Thermo Fisher Scientific) for magenta stainings. The slides were counterstained with hematoxylin (Vector Laboratories) and mounted with Roti®-Histokitt II (Carl Roth, Germany). Images were captured on an Axioplan2 imaging microscope (Carl Zeiss) equipped with an AxioCamHRc Camera (Carl Zeiss) or slides were scanned with a Vectra Polaris imaging system (PerkinElmer, Hopkinton, MA, USA) and quantified by ImageJ software.

### Chromatin immunoprecipitation

Chromatin immunoprecipitation in DLD-1 pRTR-*AP4*-VSV cells was performed according to instruction provided in the iDeal ChIP-qPCR kit (Diagenode, Belgium). The sequence information of qChIP primers is provided in Table S5.

### Micronucleus assessment

Cells were washed twice with HBSS and then fixed in 4% PFA at room temperature for 30 min. After fixation, cells were incubated with 1 μg/ml 4′6-diamidino-2-phenylindole (DAPI, Sigma) dissolved in HBSS for 5 min. Then cells were covered with ProLong™ Gold Antifade (Thermo Fisher). In order to reveal cell margins filamentous actin (F-actin) was stained with phalloidin (Alexa Fluor™ 488 Phalloidin, Thermo Fisher) at 1:50 dilution in 1% BSA. Micronuclei were microscopically determined as rounded chromatin fragments located adjacent to nuclei with a diameter not exceeding one third of the diameter of the neighboring nucleus. Microscopic analysis was performed by using a LSM 700 system (Zeiss) equipped with 63x Plan-Apochromat oil-immersion lens and ZEN 2009 software (Zeiss). For quantification, three different microscope fields were selected, and in each field, a total 50–100 cells were counted.

### Mitotic spreads and chromosome enumeration

1 × 10^7^ cells were seeded to a T-75 flask to grow 24–48 h until they reached a confluence of 90%. Cells were treated with Colcemid at a final concentration of 0.02 μg/ml for 4 h. After incubation, cells were detached by 0.5 ml 0.05% trypsin and collected in a 15 ml falcon at 160 g for 10 min. The cell pellet was resuspended in 0.5 ml of the remaining supernatant and incubated in 5 ml 0.075 M KCl solution for 30 min at room temperature. After centrifugation the pellet was resuspended in methanol-glacial acetic acid (3:1) solution. This was repeated 3 times. A small volume (5–10 μl) of cell suspension was dropped on a slide vertically using a Pasteur pipette and air dried. Chromosomes were stained by 1 μg/ml DAPI and embedded in ProLong™ Gold Antifade (Thermo Fisher). Chromosome spreading was evaluated and documented using LSM 700 system (Zeiss) equipped with 63x Plan-Apochromat oil-immersion lens. For quantification, a total 50 cells were counted in each condition.

### MTT assay

Cell viability was measured using a MTT assay. Briefly, cells were seeded in 96-well plates at 3 × 10^3^ cells/well. Before treatment with 5-FU, cells were transfected with indicated siRNAs, miRNA mimic and vectors. After 48 h, cells were treated with different doses of 5-FU for 48 h. Then 10 μl MTT solution was added per well at the concentration of 0.5 μg/μl for 4 h. The resulting Formazan was dissolved in DMSO. After agitation of the plate the absorbance was measured at 570 nm by a Varioscan system (Thermo Fisher).

### Colony formation assay

Cells were seeded into 6-well plates and transfected with indicated siRNAs, miRNA mimic and vectors for 24 h. Next, 10 μM of 5-FU was added into medium and cells were continuously cultivated for another 48 h. After finishing treatments, cells were resuspended and seeded into 12-well plates at a density of 1000 cells/well without 5-FU treatment. Colony formation was determined after 3 weeks. Colonies were recorded by a digital camera (Nikon, Japan) and enumerated by using Image J software.

### Apoptosis detection with Annexin V

Apoptosis was determined by flow cytometry after staining with Annexin V-FITC (apoptotic cell marker) and PI (necrotic cell marker) with the Annexin V-FITC/PI staining kit (556,570; BD Pharmingen, San Diego, CA, USA) according to manufacturer’s instructions. In brief, cells were harvested by addition of 0.05% trypsin (EDTA free) and washed 3 times with 1 x HBSS (Gibco, USA). Then cells were resuspended in 1 x binding buffer (0.01 M HEPES/NaOH [pH 7.4], 0.14 M NaCl, 2.5 mM CaCl_2_) at a density of 1 x 10^6^ cells/ml. 100 μl of cell suspension (1 x 10^5^ cells) was incubated with 5 μl of FITC Annexin V and 5 μl of PI for 15 min at room temperature in the dark. Before flow cytometry (CFlow6; Accuri, Ann Arbor, MI), another 400 μl of 1 x binding buffer was added to each tube and the samples were analyzed within 1 hour. Apoptotic cells were determined using the BD Accuri C6 Plus software template (BD Biosciences) with FL1-H (Annexin V-FITC) and FL3-H (PI).

### Assessment of proliferation by real-time impedance measurement

Cell proliferation was evaluated using impedance measurements (Xcelligence RTCA DP, Roche). Cells were seeded at a density of 3 x 10^3^ cells per E-plate well and subjected to the indicated treatments. Impedance was recorded every 60 min for a period of up to 120 h. A dimension-less parameter named cell-index was used to represent the electric impedance. The calculations were performed by the RTCA software integrated in the Xcelligence system. To validate impedance measurements, cells were also seeded into 96-well plates in triplicates and counted at the end time point using a Neubauer-chamber.

### Assessment of nascent RNA

To monitor de novo RNA synthesis the amount of nascent RNA was determined using the Click-iT™ Nascent RNA Capture Kit (C10365, Thermo Fisher) followed by qPCR analysis. Briefly, cells were seeded into a 12-well plate and labeled by 0.2 mM 5-ethynyl uridine (EU) for 12 h. Labeled RNA was isolated from cells by using the High Pure RNA Isolation Kit (Roche). 500 ng of labeled RNA was used for click reaction with biotin-azide, and then the reaction system was incubated with streptavidin T1 beads and washed 5 times with the provided wash-buffers. Bead-coupled RNAs were used for cDNA synthesis with the Verso cDNA Synthesis Kit (Thermo Fischer). cDNA was subjected to qPCR analysis.

### 3′-UTR dual reporter assay

The full length human *MDC1* 3’-UTR was PCR-amplified from cDNA obtained from DLD-1 cells. The PCR product was cloned into pGL3-control-MCS. To delete the miR-22-3p seed-matching sequence (SMS) in the *MDC1* 3′-UTR a QuikChange II XL Site-Directed Mutagenesis Kit (Stratagene, San Diego, CA, USA) was used according to the manufacturer’s instructions. The miR-22-3p complementary sequence (miR-22-3p antisense) was cloned as complementary DNA oligonucleotides into pGL3-control-MCS. All plasmids were verified by Sanger sequencing. The oligonucleotides used for cloning and mutagenesis are listed in Table S6. For the dual reporter assays, DLD-1 cells were seeded into a 12-well plate at 3 × 10^4^ cells/well and cultivated overnight. Transfections were performed using HiPerFect Transfection Reagent (Qiagen), 100 ng of indicated reporter vectors, and 20 ng Renilla plasmid as normalization control. After 48 h incubation with the indicated treatments, luciferase activity was measured with a Dual Luciferase Reporter assay kit (Promega) according to manufacturer’s instructions using an Orion II Microplate Luminometer (Berthold, Germany) and the Simplicity software package.

### Homologous recombination assay

Homologous recombination (HR) activity was assessed as described previously [14]. Briefly, DLD-1 and SW480 cells were transfected with pDR-GFP and pCBAScel vectors (kind gifts from Maria Jasin (Memorial Sloan Kettering Cancer Center, NY, USA) using lipofectamine LTX (Invitrogen), and co-transfected with the indicated oligonucleotides or/and plasmids. To normalize for transfection efficiency, cells were co-transfected with pcDNA3.1-mCherry (RFP). After 72 h, the percentage of GFP-expressing cells among RFP-positive cells was quantified by flow cytometry (CFlow6; Accuri).

### Bioinformatics analysis of online databases

Expression data from tumor samples and normal mucosa for relevant mRNAs was obtained and analyzed from TCGA-COAD downloaded from the National Cancer Institute’s Genomic Data Commons (https://gdc.cancer.gov/) and NCBI GEO (www.ncbi.nlm.nih.gov/geo) [15]. Expression data from colorectal cancer cell lines were obtained from the Cancer Cell Line Encyclopedia (CCLE) [16, 17]. PDX RNA expression data were obtained from GSE76402 [18]. Association of tumor samples with CMS categories was obtained and analyzed from the Cancer Subtyping Consortium (CRCSC) at www.synapse.org. AP4 ChIP-seq data from human COLO-320 cells and murine B cells, as well as c-MYC ChIP-seq data from LOVO cells were obtained from the Cistrome Data Browser (http://cistrome.org/db/#/). Expression and clinical data of the GSE14333 cohort was downloaded from NCBI GEO. The statistics for Kaplan-Meier survival curves was calculated by log-rank test. For binary classification of cases (high/low expression), the Survminer R-package (https://CRAN.R-project.org/package=survminer) was used to determine optimal cutoff values.

### Statistics

Significant differences between two groups were calculated and analyzed by a Student’s t test (two-tailed; unpaired). For multiple group comparisons, we performed 1-way analysis of variance followed by a Tukey multiple comparisons post hoc test. For gene expression associations, Pearson’s correlation was employed. *P* values less than 0.05 were considered as significant differences (*p < 0.05, **p < 0.01, ***p < 0.001, ****p < 0.0001). Statistics were performed with Prism 8 (GraphPad Software, USA).

## Results

### Generation and characterization of *AP4*-deficient CRC cell lines

**Fig. 1:**
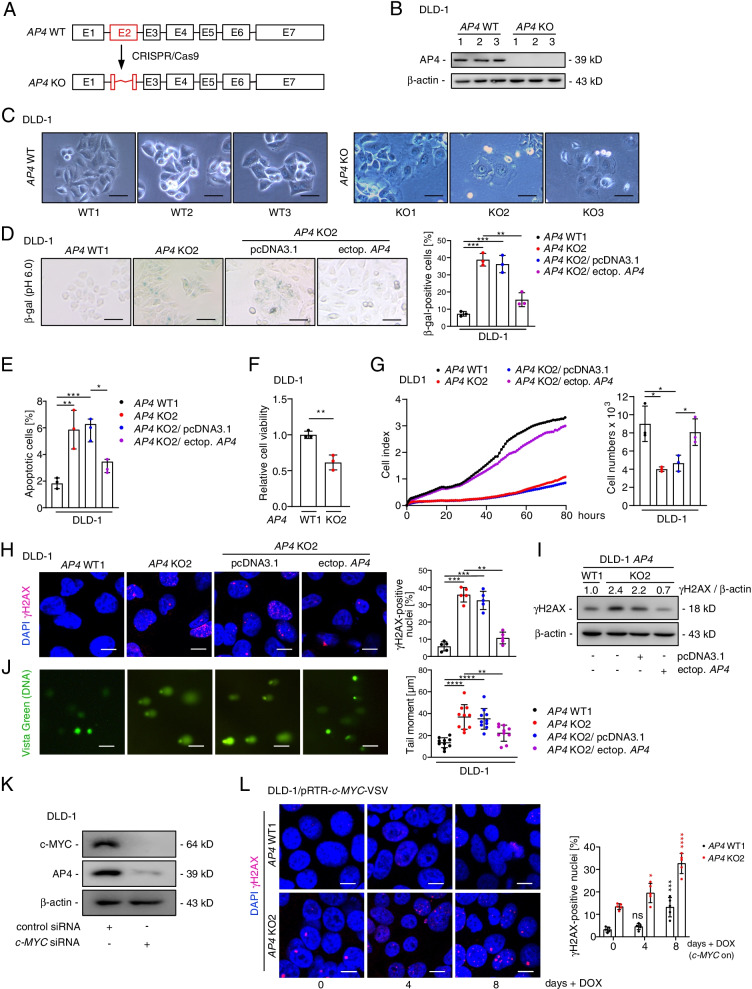
*AP4* inactivation induces DNA damage and senescence in CRC cells. **A** Scheme of targeting exon 2 (shown in red) of *TFAP4/AP4* using CRISPR/Cas9. **B** AP4 detection by Western blot analysis. β-actin served as a loading control. **C** Phase contrast images of untreated cells. Scale bars: 50 μm. **D** Detection of senescent cells using pH 6 β-gal staining 48 h after transfection. Ectopic expression of AP4 was achieved using a pcDNA-*AP4*-VSV vector described in [4]. Three fields of 120 cells in total were evaluated. Scale bars: 100 μm. **E** Quantification of apoptotic cells by Annexin V detection 48 h after transfection. **F** MTT assay results obtained 72 h after seeding. **G** Proliferation was determined by impedance measurement in E-plates 48 h after transfection in the indicated cells (left panel). Cell numbers were determined at the last time point (right panel). **H** Detection of γH2AX foci 48 h after transfection. Quantification of 5 fields with 150 cells in total. Scale bars: 20 μm. **I** Western blot analysis 48 h after transfection. **J** Comet assay 48 h after transfection. Quantification of DNA tail moment in 10 fields with 150 cells in total. Scale bars: 10 μm. **K** Western blot analysis after 48 h after c-MYC siRNA transfection. **L** γH2AX foci detection after *c-MYC* induction by addition of DOX. Quantification of 5 fields with 150 cells in total. Scale bars: 20 μm. Results are presented as the mean + SD with (*n* = 3) for **D-G**, (*n*=5) for **H+L ** and (*n*=10) for **J** with *: *p* < 0.05, **: *p* < 0.01, ***: *p* < 0.001

In order to generate *AP4*-deficient colorectal cancer cell lines, DLD-1 and SW480 cells were transfected with pSp-Cas9-GFP vectors expressing three guide RNAs targeting exon 2 of the *TFAP4/AP4* gene, which encodes the DNA binding region of AP4 (Fig. 1A). Three *AP4*-deficient single cell derived clones were obtained for each cell line that showed a lack of AP4 protein expression (Fig. 1B and Fig. S1A). Interestingly, *AP4*-deficient DLD-1 and SW480 were often enlarged and flattened (Fig. 1C and Fig. S1B) and the number of cells positive for senescence-associated β-galactosidase at pH 6 (SA-β-gal) was significantly increased in *AP4*-deficient versus *AP4*-proficient DLD-1 cells. Ectopic expression of *AP4* reduced the number of cells positive for SA-β-gal in *AP4*-deficient DLD-1 cells (Fig. 1D), confirming that the observed effect is due to loss of AP4 function. Therefore, AP4 suppresses senescence in CRC cells. In addition, the colony-forming capacity was decreased in *AP4*-deficient DLD-1 and SW480 cells (Fig. S1C). The frequency of apoptotic cells was increased in *AP4-*deficient DLD-1 and SW480 cells when compared to *AP4*-proficient cells. Again, ectopic expression of *AP4* reversed this effect (Fig. 1E and Fig. S1E). Furthermore, the viability of *AP4*-deficient DLD-1 and SW480 cells was significantly lower when compared to *AP4*-proficient cells as determined in an MTT assay (Fig. 1F and Fig. S1F). *AP4*-deficient DLD-1 and SW480 cells also displayed a pronounced decrease in proliferation. Ectopic *AP4* largely reverted the decreased proliferation of *AP4*-deficient DLD-1 and to a lesser degree that of *AP4*-deficient SW480 cells (Fig. 1G and Fig. S1G). The proliferation defect caused by *AP4* deficiency observed here is presumably largely due the induction of senescence. Since senescence is known to result from DNA damage we evaluated the amount of spontaneous DNA damage. Indeed, untreated *AP4*-deficient DLD-1 cells displayed elevated levels of endogenous DNA damage as evidenced by a significant increase in γH2AX-positive foci and γH2AX protein levels when compared to *AP4*-proficient DLD-1 cells (Fig. 1H, I). The increase in γH2AX-positive foci and γH2AX levels in AP4-deficient cells was reversed by ectopic expression of *AP4*. In a comet assay, *AP4*-deficient DLD-1 cells showed a longer tail of unrepaired, damaged DNA when compared to *AP4*-proficient DLD-1 cells (Fig. 1J), suggesting that *AP4*-deficiency causes a defect in DNA repair. Ectopic expression of *AP4* in *AP4*-deficient DLD-1 cells suppressed DNA damage. A similar effect of *AP4*-deficiency on DNA damage was observed in *AP4*-deficient SW480 cells (Fig. S2A-C). We have previously shown that expression of AP4 is directly induced by c-MYC in breast cancer cells, human diploid fibroblasts and SW620 CRC cells [4, 19]. In line with these findings, down-regulation of c-MYC by specific siRNAs resulted in a decrease in AP4 expression in DLD-1 and SW480 cells (Fig. 1K and Fig. S2D). Furthermore, activation of a conditional *c-MYC* allele by addition of DOX for up to 8 days resulted in more DNA damage in *AP4*-deficient than in *AP4*-proficient CRC cells (Fig. 1L and Fig. S2E-F). Taken together, the results indicate that AP4 suppresses spontaneous and c-MYC-induced DNA damage in CRC cells. This may be due to role of AP4 in facilitating the repair of DNA damage*.* Therefore, the suppression DNA of damage by AP4 may decrease the rates of senescence and apoptosis in CRC cells displaying enhanced expression of c-MYC.

### AP4 directly represses *MIR22HG*

**Fig. 2:**
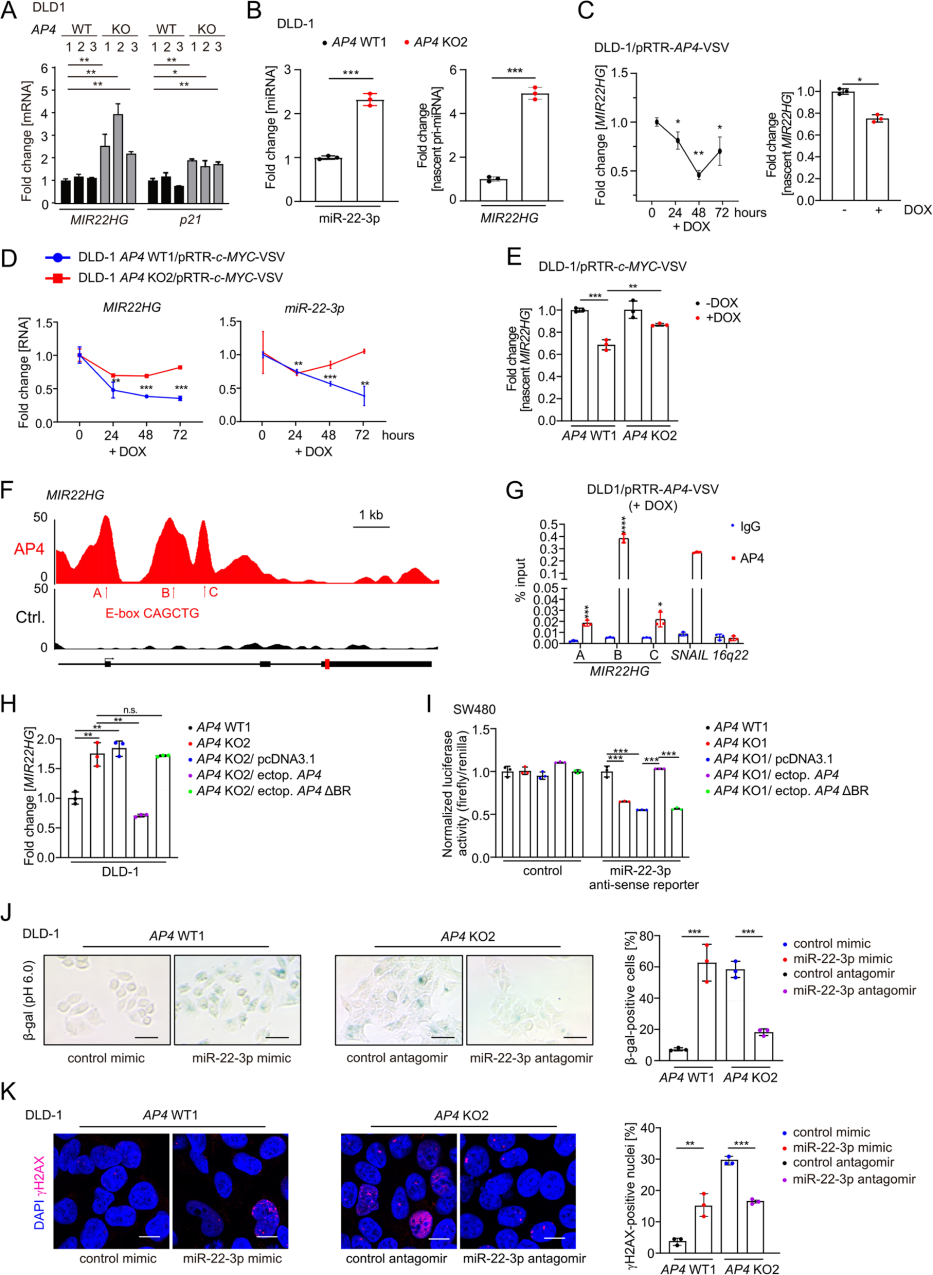
AP4 directly represses *MIR22HG*. **A** qPCR analysis of *MIR22HG* and *p21* in the indicated clones of DLD-1 cells. **B** qPCR analysis of miR-22-3p expression (left panel) and nascent *MIR22HG* mRNA (right panel). **C** qPCR analysis of *MIR22HG* expression after AP4 induction by DOX for the indicated duration (left panel) and nascent *MIR22HG* 48 h after AP4 induction by DOX (right panel). **D** qPCR analysis of *MIR22HG* (left panel) and miR-22-3p (right panel) after c-MYC induction by DOX for indicated durations. **E** qPCR analysis of nascent *MIR22HG* 48 h after c-MYC induction by DOX. **F** ChIP-Seq enrichment profiles for *AP4*-VSV associated chromatin were generated with the UCSC genome browser. The gene structure ideogram is shown below the ChIP-seq tracks. **G** qChIP analysis of AP4 occupancy. Cells were treated with DOX for 48 h. Chromatin was enriched by anti-AP4 or anti-rabbit-IgG antibodies. *SNAI1* and *16q22* served as positive and negative controls, respectively. **H** qPCR analysis of *MIR22HG* in the indicated cells 48 h after transfections. **I** miR-22-3p anti-sense reporter dual-luciferase analysis 48 h after transfection. **J** β-gal detection at pH 6 48 h after transfection. Quantification of 3 fields with 120 cells in total. Scale bars: 50 μm. **K** Detection of γH2AX foci 48 h after transfection. Quantification of 3 fields with 120 cells total. Scale bars: 20 μm. In panel **A**-**E** and **G**-**K** the mean + SD (*n* = 3) is provided with *: *p* < 0.05, **: *p* < 0.01, ***: *p* < 0.001, ****: *p* < 0.0001

Next, we comprehensively determined the effects of *AP4* deletion on global mRNA expression by RNA-Seq analysis (Kaller et al., in preparation). Among the genes up-regulated after *AP4* deletion in DLD-1 cells was *CDKN1A/p21* (Fig. 2A), which we had previously identified as a direct AP4 target gene [4]. Notably, the long non-coding/lncRNA *MIR22HG* was also up-regulated in *AP4*-deficient CRC cells (Fig. 2A). Interestingly, the *MIR22HG*-derived microRNA miR-22-3p is known to induce premature senescence [20]. An *AP4*-dependent regulation of *CDKN1A/p21* and *MIR22HG* was also detected in SW480 cells (Fig. S3A). The expression of miR-22-3p was also elevated in *AP4*-deficient CRC cells (Fig. 2B). Also the amount of newly synthesized, nascent *MIR22HG* mRNA was increased in *AP4*-deficient DLD-1 and SW480 cells (Fig. 2B and Fig. S3B), suggesting a direct effect of AP4 on the transcription of *MIR22HG*. Furthermore, the amount of total and nascent *MIR22HG* mRNA was decreased after ectopic expression of *AP4* from an episomal pRTR vector in DLD-1 cells (Fig. 2C). Also after ectopic expression of c-MYC, the expression of *MIR22HG* and miR-22-3p and that of nascent *MIR22HG* was repressed in an *AP4*-dependent manner in DLD-1 and SW480 cells (Fig. 2D-E and Fig. S2C- E). The known AP4 targets, *SNAI1* and *CDKN1A/p21* were also regulated in an *AP4*-dependent manner after activation of ectopic c-MYC in these cells (Fig. S3F). We detected AP4 occupancy at the *MIR22HG* promoter by ChIP-Seq analysis (Fig. 2F; Kaller et al., in preparation). Since we also identified E-box motifs (CAGCTG), which represent the binding sites of AP4, under the ChIP-Seq peaks, AP4 presumably binds directly to the promoter of *MIR22HG* (Fig. 2F). These results were confirmed by qChIP analysis (Fig. 2G). The increased expression of *MIR22HG* in *AP4*-deficient CRC cells was reverted by ectopic expression of *AP4*, whereas a mutant *AP4* lacking its basic region (*AP4* ΔBR), described in [4], had no effect (Fig. 2H and Fig. S3G). Therefore, the repression of *MIR22HG* requires the DNA binding region of AP4 and occurs by direct binding of AP4 to the *MIR22G* promoter. In addition, the levels of functional miR-22-3p were repressed by ectopic *AP4* in *AP4*-deficient SW480 cells, as determined by a luciferase assay using a miR-22-3p antisense reporter (Fig. 2I). This effect was also dependent on the DNA-binding ability of AP4. Therefore, AP4 directly represses *MIR22HG* and thereby leads to a functionally relevant decrease of miR-22-3p.

Since miR-22-3p has been implicated in senescence of human fibroblasts and breast cancer cells before [20], we analyzed whether miR-22-3p plays a role in the cellular senescence caused by *AP4*-deficiency. After transfection of DLD-1 *AP4* WT1 cells with a miR-22-3p mimic an increase in SA-β-gal positive cells was observed, while inhibition of miR-22-3p by a specific antagomir reduced cellular senescence in DLD-1 *AP4* KO2 cells (Fig. 2J). Similar effects on senescence were detected in SW480 cells (Fig. S3H). The degree of senescence caused by ectopic miR-22-3p expression was similar to the effect of *AP4* deletion, indicating that up-regulation of miR-22-3p may mediate senescence observed after deletion of *AP4*. Ectopic miR-22-3p also elevated the amount of DNA damage in the DLD-1 *AP4* WT1 clone (Fig. 2K). Conversely, inhibition of miR-22-3p by an antagomir reduced DNA damage as evidenced by a reduction in γH2AX-positive foci in DLD-1 *AP4* KO2 cells. Since miR-22-3p expression was sufficient and required for increased senescence and DNA damage in DLD-1 and SW480 cells, the up-regulation of miR-22-3p caused by AP4 inactivation in CRC cells presumably mediates a significant portion of the observed increase in DNA damage and cellular senescence.

### *MDC1* is a direct target of AP4

**Fig. 3:**
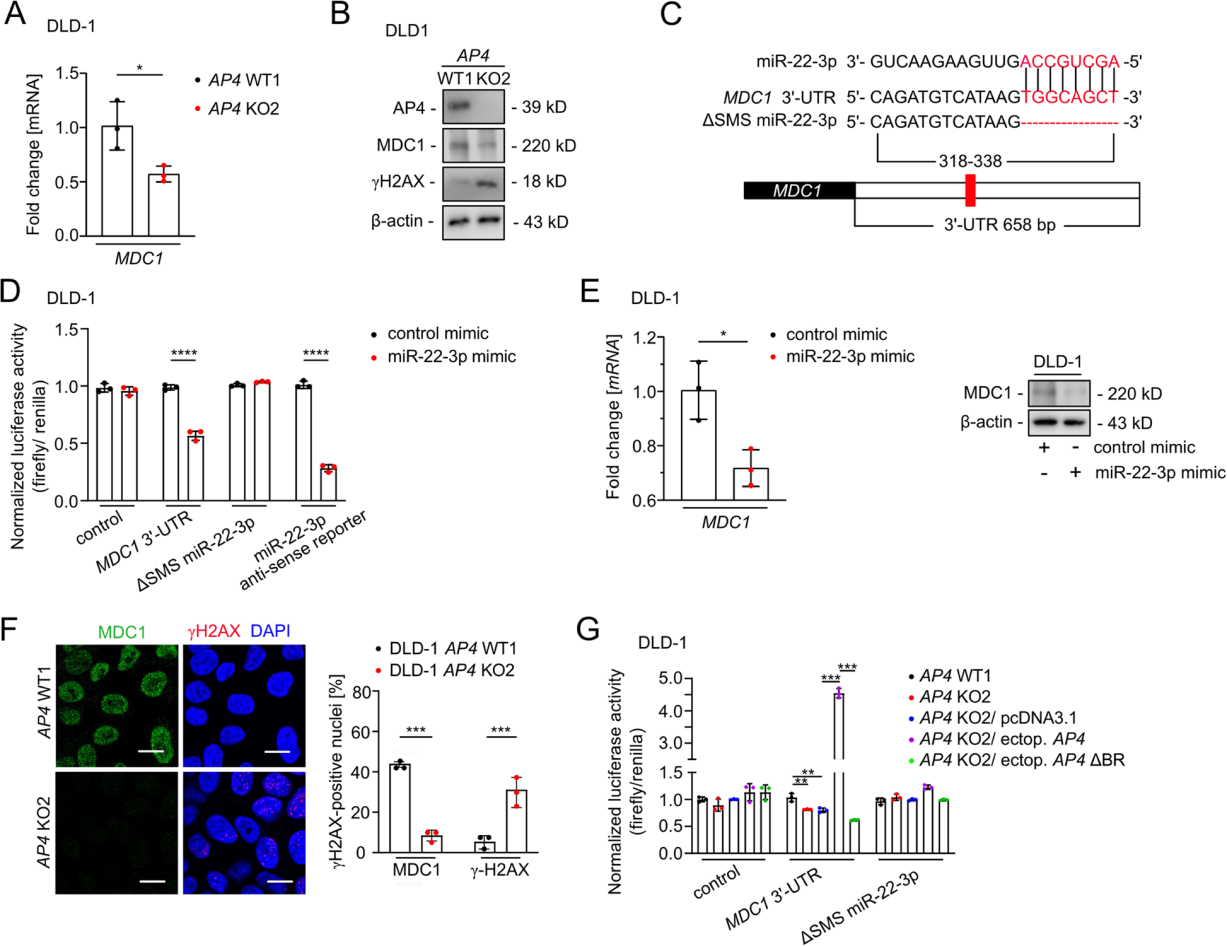
AP4-mediated repression of miR-22-3p contributes to increased MDC1 levels. **A** qPCR analysis of *MDC1* expression. **B** Western blot analysis. **C** Scheme of the miR-22-3p seed, the seed-matching sequences and its deletion in the 3′-UTR in the *MDC1* mRNA. The seed and seed-matching sequences are highlighted in red. **D** Dual-luciferase assay was conducted 48 h after DLD-1 cells were transfected with miR-22-3p mimic and human *MDC1* 3′-UTR reporter vector. The miR-22-3p anti-sense reporter served as a positive control. **E** qPCR (left panel) and Western blot analysis (right panel) 48 h after transfection. **F** MDC1 foci detection in untreated cells. Quantification of 3 fields with 120 cells in total. Scale bars: 20 μm. **G** Dual-luciferase assay 48 h after transfection. In panels **A**, **D**-**G**, the mean + SD (*n* = 3) is provided with *: *p* < 0.05, **: *p* < 0.01, ***: *p* < 0.001, ****: *p* < 0.0001

Among the known miR-22-3p targets [21], *MDC1/Mediator of DNA damage Checkpoint 1* appeared to be most relevant in this context, since it is a central component of the DNA damage response [22–24]. Therefore, we asked whether down-regulation of MDC1 expression occurs via the elevated miR-22-3p expression characteristic for *AP4*-deficient CRC cells. Indeed, the amount of MDC1 mRNA and protein was decreased in *AP4*-deficient DLD-1 and SW480 cells (Fig. 3A-B and Fig. S4A-B). We confirmed that *MDC1* mRNA is a direct target of miR-22-3p in DLD-1 cells in a dual reporter assay (Fig. 3C-D). Furthermore, ectopic miR-22-3p expression decreased MDC1 mRNA and protein levels in DLD-1 and SW480 cells (Fig. 3E and Fig. S4C-D). On the cellular level, more MDC1-positive and less γH2AX-positive foci were detected in the nuclei of *AP4*-proficient DLD-1 and SW480 cells, when compared to *AP4*-deficient cells (Fig. 3F and Fig. S4E). In order to determine whether the repression of *MIR22HG*/miR-22-3p by AP4 is relevant for the regulation of *MDC1* transcript levels we performed a reporter assay (Fig. 3G). Indeed, ectopic expression of AP4 in *AP4*-deficient cells induced the activity of the *MDC1*–3′-UTR reporter in a manner dependent on the miR-22-3p SMS and the DNA binding capacity of AP4. Therefore, the repression of *MIR22HG* by AP4 may increase the abundance of *MDC1* mRNA.

**Fig. 4:**
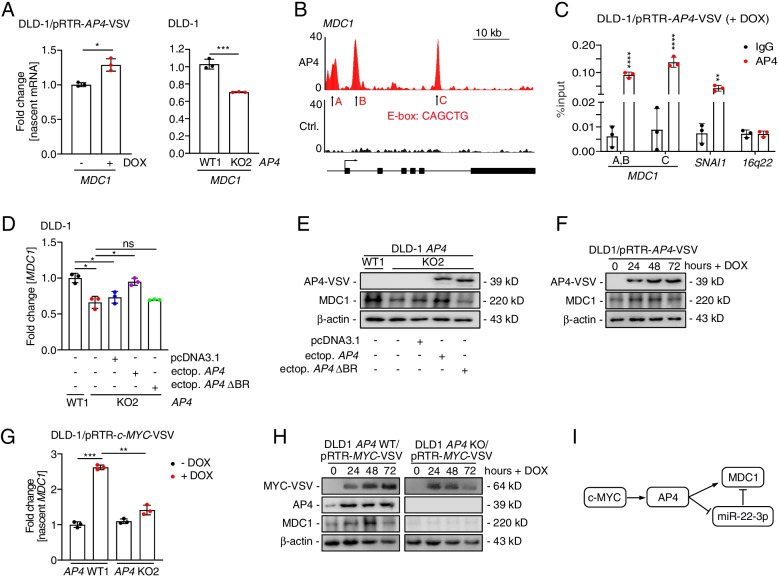
MDC1 is directly regulated by AP4. **A** qPCR analysis of nascent *MDC1* mRNA 48 h after AP4 induction by DOX (left panel). qPCR analysis of nascent *MDC1* mRNA in untreated cells (right panel). **B** ChIP-Seq analysis of AP4 occupancy using the UCSC genome browser. **C** Validation of *MDC1* as AP4 direct target via qChIP assay. Cells were treated with DOX for 48 h. Chromatin was enriched by anti-AP4 or anti-rabbit-IgG antibodies. *SNAI1* and *16q22* served as positive and negative controls, respectively. **D**-**E** qPCR and Western blot analysis of MDC1 in the indicated cell lines 48 h after transfection. **F** Western blot analysis of MDC1 after AP4 induction by DOX for the indicated durations. **G** qPCR analysis of nascent *MDC1* mRNA after c-MYC induction by DOX for 48 h. **H** Western blot analysis after c-MYC induction by DOX for the indicated periods. **I** Model of the *AP4*/miR-22-3p/*MDC1* coherent feed-forward loop. In panels **A**, **C**, **D** and **G**, the mean + SD (*n* = 3) is provided with *: *p* < 0.05, **: *p* < 0.01, ***: *p* < 0.001, ****: *p* < 0.0001

In addition, ectopic *AP4* increased the amount of nascent *MDC1* mRNA and *AP4*-deficient cells showed a decrease in nascent *MDC1* mRNAs (Fig. 4A and Fig. S4F), suggesting that AP4 may also directly induce *MDC1* transcription. Indeed, an E-box motif upstream of the TSS and two E-box motifs in the introns of *MDC1* showed occupancy by AP4 in DLD-1 cells according to a ChIP-Seq analysis (Fig. 4B; Kaller et al., in preparation). The AP4 occupancy of these binding sites within the *MDC1* promoter was confirmed by qChIP analysis (Fig. 4C). Ectopic AP4 expression resulted in increased *MDC1* expression in *AP4*-deficient DLD-1 and SW480 cells, whereas ectopic *AP4* ΔBR was unable to induce *MDC1* in *AP4*-deficient DLD-1 and SW480 cells (Fig. 4D-E, and Fig. S4G-H). Furthermore, the expression of MDC1 protein was induced by ectopic *AP4* in DLD-1 cells (Fig. 4F). c-MYC activation also induced *MDC1* transcription and MDC1 protein expression in an *AP4*-dependent manner in DLD-1 and SW480 cells (Fig. 4G-H and Fig. S4I-J). Taken together these results show that after c-MYC activation AP4 induces *MDC1* expression directly and indirectly via repressing miR-22-3p (Fig. 4I). This type of dual regulation, also termed coherent feed-forward loop, is known to confer robustness to transcriptional networks [25].

### Associations of *c-MYC*, *AP4*, *MDC1* and miR-22-3p expression in primary CRCs

**Fig. 5:**
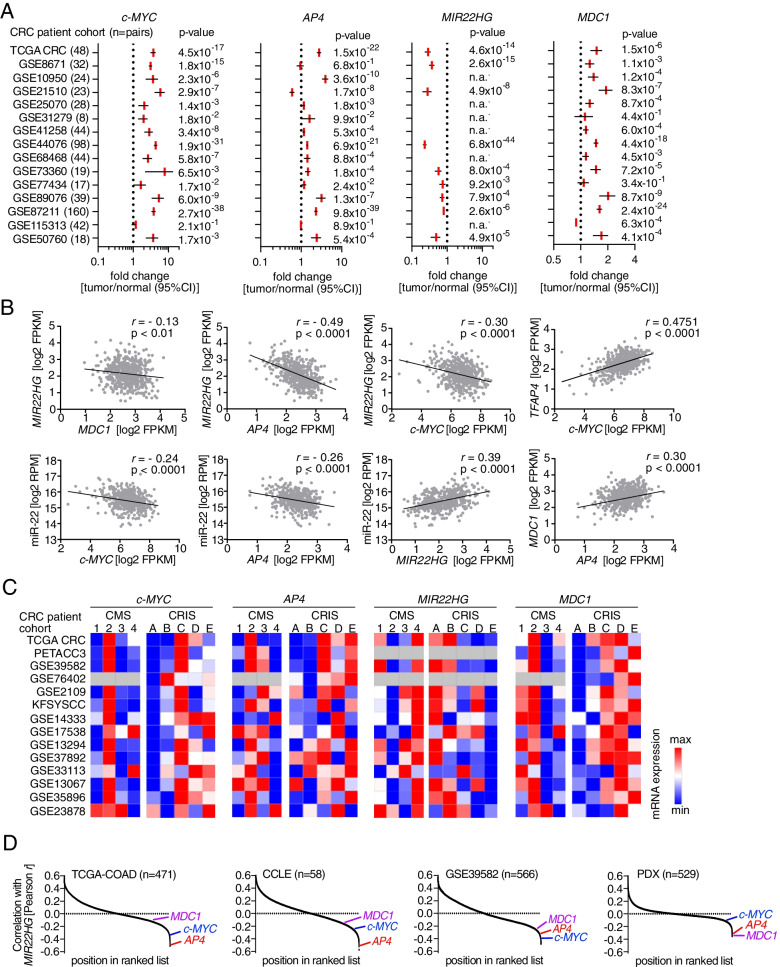
Correlations between *c-MYC*, *AP4*, *MDC1* and miR-22-3p expression in primary CRCs. **A** Relative expression of the indicated mRNAs detected in expression profiles from patient-derived normal mucosa and tumor tissue deposited in the TCGA-COAD and GEO databases. **B** Scatter plots of pair-wise comparisons of mRNA expression of the indicated genes in TCGA-COAD samples. **C** Heat maps of associations between *c-MYC*, *AP4*, *MIR22HG* and *MDC1* expression and CMS/CRIS subtypes in the indicated patient cohorts. **D** RNAs were ranked in descending order according to Pearson correlation coefficient r with *MIR22HG* expression for the indicated datasets. The positions of *MDC1*, *c-MYC* and *AP4* expression in the ranked lists are indicated

To determine whether the regulation of *MIR22HG/miR-22-3p* and *MDC1* by *AP4* also occurs in primary CRCs, we analyzed the expression of these genes in 15 cohorts of colorectal adenocarcinomas (COAD) provided by the TCGA consortium and GEO. Within these datasets c-*MYC*, *AP4* and *MDC1* expression was significantly increased, whereas *MIR22HG* expression was repressed in primary CRCs obtained from 644 patients when compared to matched normal mucosa (Fig. 5A). *MIR22HG* showed a significantly negative correlation with *MDC1*, *AP4* and c-*MYC* expression in 471 primary CRC samples of the TCGA-COAD cohort (Fig. 5B). *AP4* and *c-MYC* also showed a negative correlation with miR-22-3p. In addition, the expression of *MDC1* and *c-MYC* showed a positive correlation with *AP4* in CRCs. As expected, *MIR22HG* and its product *miR-22-3p,* as well as c-*MYC* and *AP4* displayed a positive correlation in CRCs. Furthermore, c-*MYC, AP4* and *MDC1* showed the highest expression in CRCs belonging to the CMS2 subtype, whereas the expression of *MIR22HG* was lowest in CMS2 CRCs. Interestingly, CMS2 is characterized by elevated WNT and c-MYC pathway activity, as well as chromosomal instability. Since stromal cell derived mRNAs may confound the gene expression profiles of CRCs, we also performed an analysis using CRC intrinsic subtypes (CRIS). These allow tumor classification according to gene signatures obtained from patient-derived tumor xenografts in mice [18]. c-*MYC*, *AP4* and *MDC1* were mainly elevated in CRIS C-E subtypes, which are characterized by elevated EGFR, WNT signaling and *TP53* mutations. Conversely, *MIR22HG* was decreased in CRIS C-E subtypes (Fig. 5C). Of note, *MDC1*, *AP4* and *c-MYC* also displayed a negative association with *MIR22HG* expression in an independent patient cohort (GSE39582), as well as in expression data from CRC cell lines (CCLE) and in patient-derived xenografts (PDX) (Fig. 5D). Therefore, an inverse correlation between *MIR22HG*/miR-22-3p and *c-MYC/AP4/MDC1* expression is also evident in primary CRCs, implying that the regulations identified here are conserved in vivo. In summary, the expression of *c-MYC, AP4* and *MDC1* is coordinately elevated in primary CRCs, whereas *MIR22HG/miR-22-3p* is repressed. This pattern is mainly associated with CMS2 and CRIS C-E subtypes, which represent CRCs with enhanced WNT signaling.

### Regulation of *Mir22hg*/*MDC*1 by Ap4 in mice

**Fig. 6:**
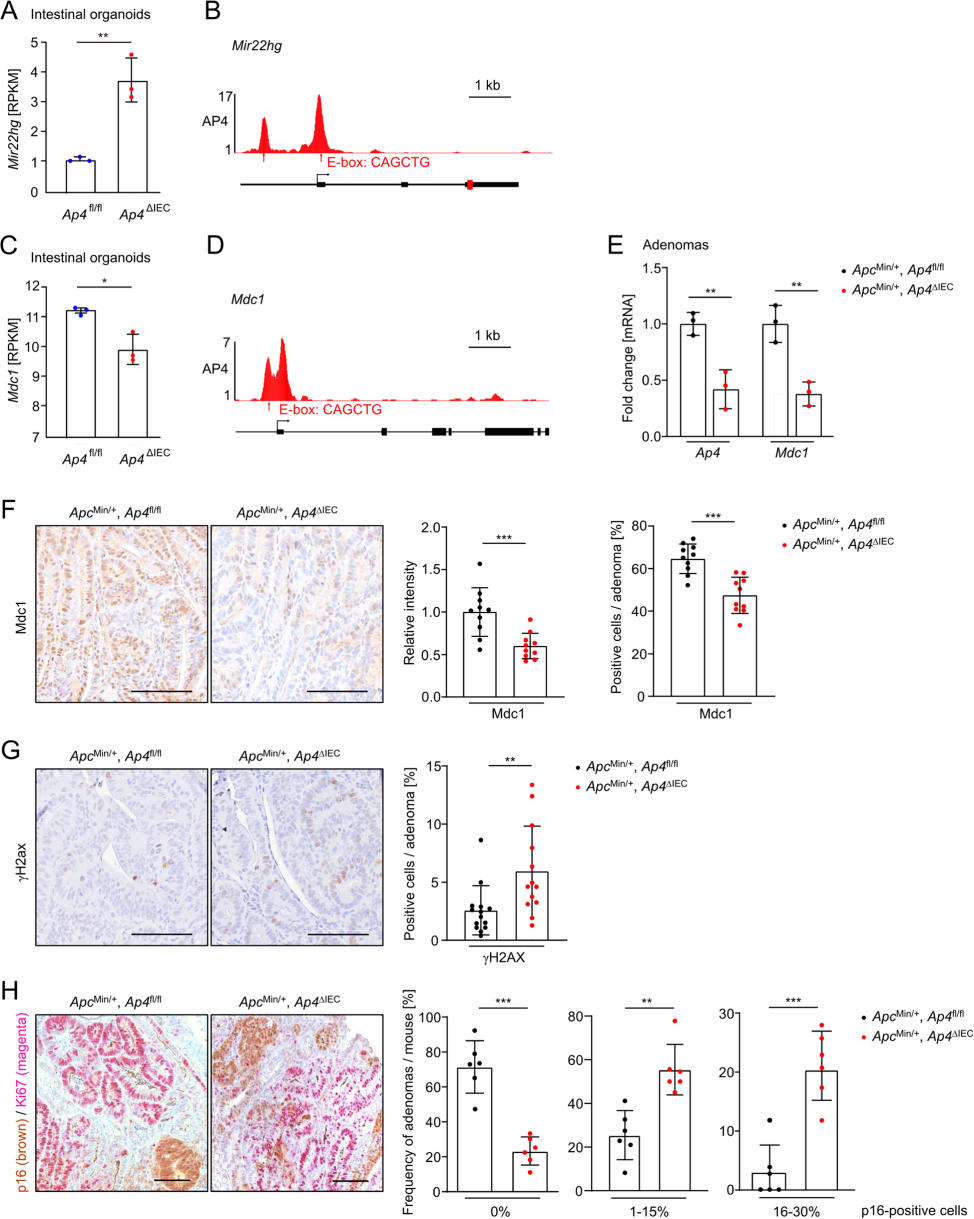
Regulation of *MIR22HG* and *MDC1* by AP4 is conserved in mice. **A** Differential expression of *Mir22hg* in *Ap4*-deficient intestinal organoids. **B+D** ChIP-Seq enrichment profiles for *Ap4*-associated chromatin were generated with the UCSC genome browser. The data was obtained from GSM2132681. The structures of the **B** *Mir22hg* and the **D** *Mdc1*genes are shown below the ChIP-Seq tracks. **C** Differential expression of *Mdc1* in *Ap4*-deficient intestinal organoids. **E** qPCR analysis of adenomas from three 120 days old *Apc*^Min/+^ mice (five adenomas per mouse) per genotype. **F** Left panel: Immunohistochemical detection of Mdc1 in adenomas of 120 days old *Apc*^Min/+^ mice with the indicated genotype. Counterstaining with hematoxylin. Scale bar = 100 μm. Right panel: Quantification of Mdc1 relative intensity and Mdc1-positive cells in percent (%) in adenomas from 120 days old *Apc*^Min/+^ mice in a total of 10 adenomas per genotype. **G** Left panel: Immunohistochemical detection of γH2AX in adenomas of 120 days old ApcMin/+ mice with the indicated genotype. Right panel: Quantification of γH2AX-positive cells in percent (%) in adenomas from 120 days old *Apc*^Min/+^, *Ap4*^fl/fl^ and *Apc*^Min/+^, *Ap4*^∆IEC^ mice in a total of 14 or 13 adenomas, respectively. **H** Left panel: Immunohistochemical detection of p16 in adenomas of 120 days old *Apc*^Min/+^ mice with the indicated genotype. Right panel: Quantification of p16-positive cells in percent (%) in adenomas from 120 days old *Apc*^Min/+^*Ap4*^fl/fl^ and *Apc*^Min/+^, *Ap4*^∆IEC^ mice in 26 or 22 adenomas, respectively. In panels **F**-**H** scale bars represent 100 μm. *:*p* < 0.05, **:*p* < 0.01, ***:*p* < 0.001

Next, we analyzed whether the regulation of miR-22-3p and *MDC1* by AP4 is conserved in adenomas from *Apc*^*Min/+*^ mice, which represent a model for inherited colorectal cancer (familial adenomatous polyposis (FAP) [26]. *Apc*^*Min/+*^ mice harbor an inactivating mutation in one *Apc* allele. Upon spontaneous loss of the second *Apc* allele, these mice develop multiple intestinal adenomas. In RNA-Seq results obtained in our previous study [6] *Mir22hg* expression was significantly increased in intestinal organoids with deletion of *Ap4* (Fig. 6A), which is in line with the results obtained in *AP4*-deficient CRC cells. Ap4 occupancy of murine *Mir22hg* (Fig. 6B)*,* which coincides with the presence of two E-box motifs, was detected in published ChIP-Seq results [27]. *Mdc1* expression was significantly decreased in *Ap4*-deficient intestinal organoids (Fig. 6C). In addition, an Ap4 E-Box binding motif displaying occupancy by Ap4 was detected in the *Mdc1* promoter region (Fig. 6D). Furthermore, a decrease in *Mdc1* mRNA expression was found in adenomas from *Apc*^Min/+^ mice with intestinal epithelial cell (IEC)-specific deletion of *Ap4* when compared to *Ap4*-wild-type *Apc*^*Min/+*^ mice (Fig. 6E). Taken together, these results imply that the regulation of *MIR22HG* and *MDC1* by AP4 is conserved in mice. In addition, a decrease in Mdc1 protein expression and in the number of Mdc1-positive cells was detected by immunohistochemistry in *Ap4*^∆IEC^ adenomas of 120 days old *Apc*^Min/+^ mice when compared to *Ap4*^fl/fl^ adenomas (Fig. 6F). Conversely, an increase in γH2AX-positive cells was detected in *Ap4*^∆IEC^ adenomas (Fig. 6G), indicating higher levels of endogenous DNA damage in *Apc*^Min/+^/*Ap4*^ΔIEC^ adenomas. Furthermore, *Ap4*^ΔIEC^ adenomas displayed an increase of cells positive for the senescence marker p16/INK4A when compared to *Ap4*^fl/fl^ adenomas (Fig. 6H). As expected, p16-positive cells were negative for the proliferation marker Ki67. Taken together, these results show that the regulation of DNA repair and senescence by AP4 via its targets *MIR22HG* and *MDC1* is conserved in mice and occurs in vivo.

### MDC1 mediates effects of AP4 on DNA damage

**Fig. 7:**
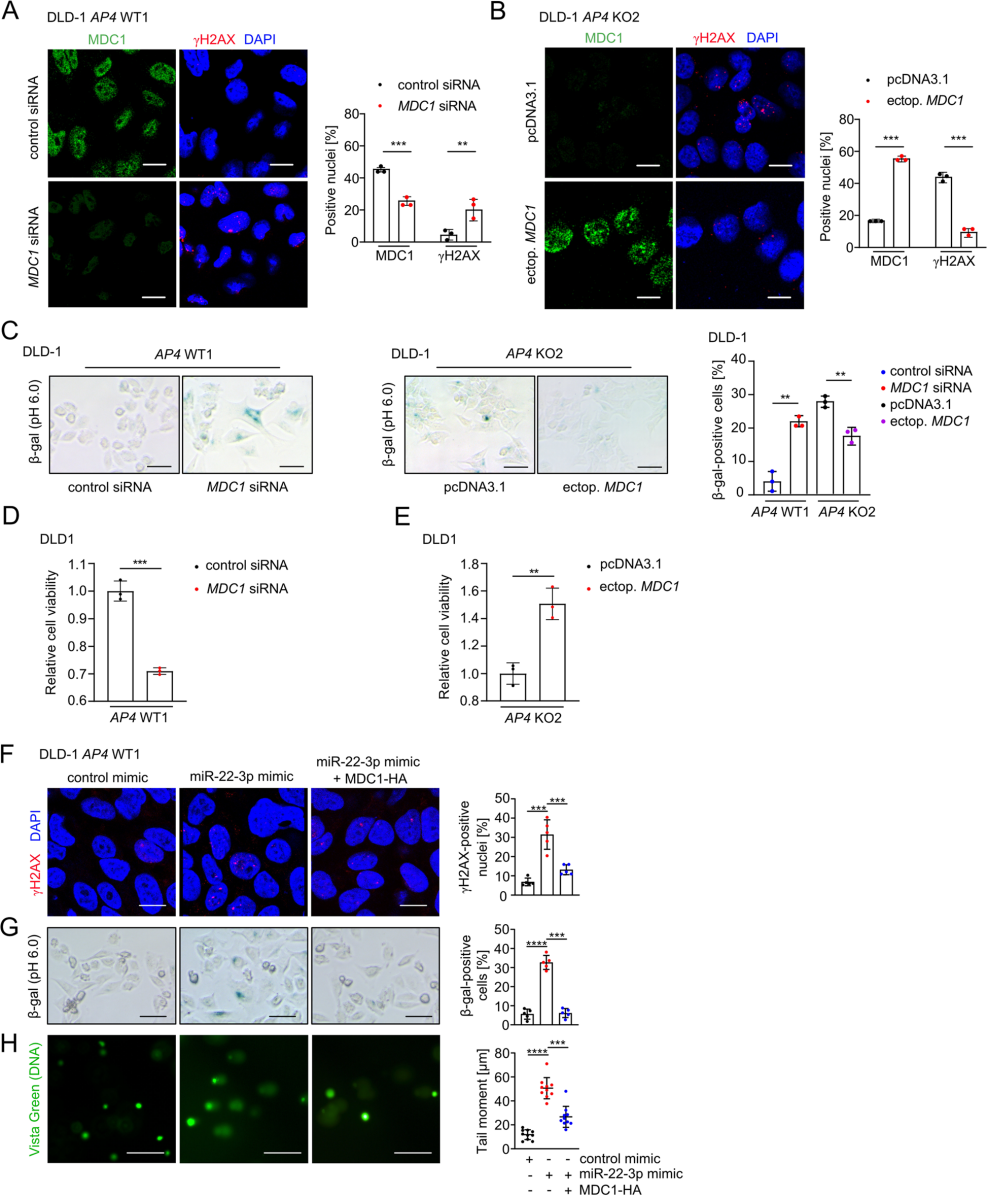
MDC1 mediates effects of AP4 on DNA repair. **A** Detection of MDC1 and γH2AX foci by immunocytochemistry 48 h after silencing MDC1. Scale bars: 20 μm. Foci quantification was performed with Image J software. Nuclei with over 10 foci were considered as positive. The fluorescence intensity was normalized to DAPI. Quantification of 3 fields with 120 cells in total. **B** MDC1 and γH2AX foci were detected by immunocytochemistry 48 h after ectopic expression of MDC1. Scale bars: 20 μm. Nuclei with over 10 foci were considered as positive. The fluorescence intensity was normalized to DAPI. Quantification of 3 fields with 120 cells in total. **C** β-gal staining 48 h after silencing or ectopic expression of MDC1, respectively. Quantification of 3 fields with 120 cells in total. Scale bars: 50 μm. MTT assay results 48 h after **D** silencing *MDC1* or **E** ectopic expression of *MDC1*. Detection of **F** γH2AX foci by immunocytochemistry, **G** β-gal staining and **H** comet assay in DLD-1 *AP4* WT1 cells 48 h after transfection. MDC1-HA was rendered non-responsive to miR-22-3p by deletion of the *MDC1* 3'-UTR [28]. Quantification of DNA tail moment in 10 fields with 150 cells in total. The mean + SD is provided with **A**-**E** (*n* = 3), **F**-**G** (*n* = 5) and **H** (*n* = 10) with **: *p* < 0.01, ***: *p* < 0.001, ****: *p* < 0.0001

Next, we determined the functional relevance of MDC1 in CRC lines with varying *AP4* status. When the expression of MDC1 was decreased using siRNAs in *AP4*-proficient DLD-1 and SW480 cells we detected an increase of cells with nuclear γH2AX foci (Fig. 7A and Fig. S5A). However, when MDC1 was ectopically expressed in *AP4*-deficient DLD-1 and SW480 cells the frequency of nuclei with detectable γH2AX foci decreased (Fig. 7B and Fig. S5B). Therefore, MDC1 is required to suppress DNA damage in AP4 expressing cells and sufficient to rescue a DNA damage repair defect in *AP4*-deficient CRC cells. Silencing of *MDC1* in *AP4*-proficient DLD-1 and SW480 cells resulted in increased cellular senescence as evidenced by increased SA-β-gal staining (Fig. 7C and Fig. S5C). Conversely, ectopic expression of MDC1 suppressed senescence in *AP4*-deficient DLD-1 and SW480 cells (Fig. 7C and Fig. S5C). Therefore, the presence of unrepaired DNA damage resulting from the modulation of MDC1 levels by AP4 correlated with the amount of cell undergoing senescence. In addition, the viability of *AP4*-proficient CRC cells was significantly lower when MDC1 was silenced by specific siRNAs (Fig. 7D and Fig. S5D), whereas ectopic MDC1 expression increased the viability of *AP4*-deficient CRC cells (Fig. 7E and Fig. S5E). To determine whether the effect of miR-22-3p on cellular senescence and DNA damage was mediated by targeting *MDC1* and not another miR-22-3p target mRNA, we co-expressed miR-22-3p and a miR-22-3p-insensitive *MDC1* mRNA in *AP4*-proficient DLD-1 and SW480 cells (Fig. 7F-H and Fig. S5F-H). Indeed, expression of the miR-22-3p-insensitive *MDC1* largely alleviated the DNA damage and cellular senescence caused by miR-22-3p. Taken together, AP4 suppresses DNA damage by inducing MDC1, which facilitates DNA repair. As a consequence cellular senescence is decreased and viability increased in cells expressing AP4.

### MDC1 mediates effects of AP4 on CIN and HR

**Fig. 8:**
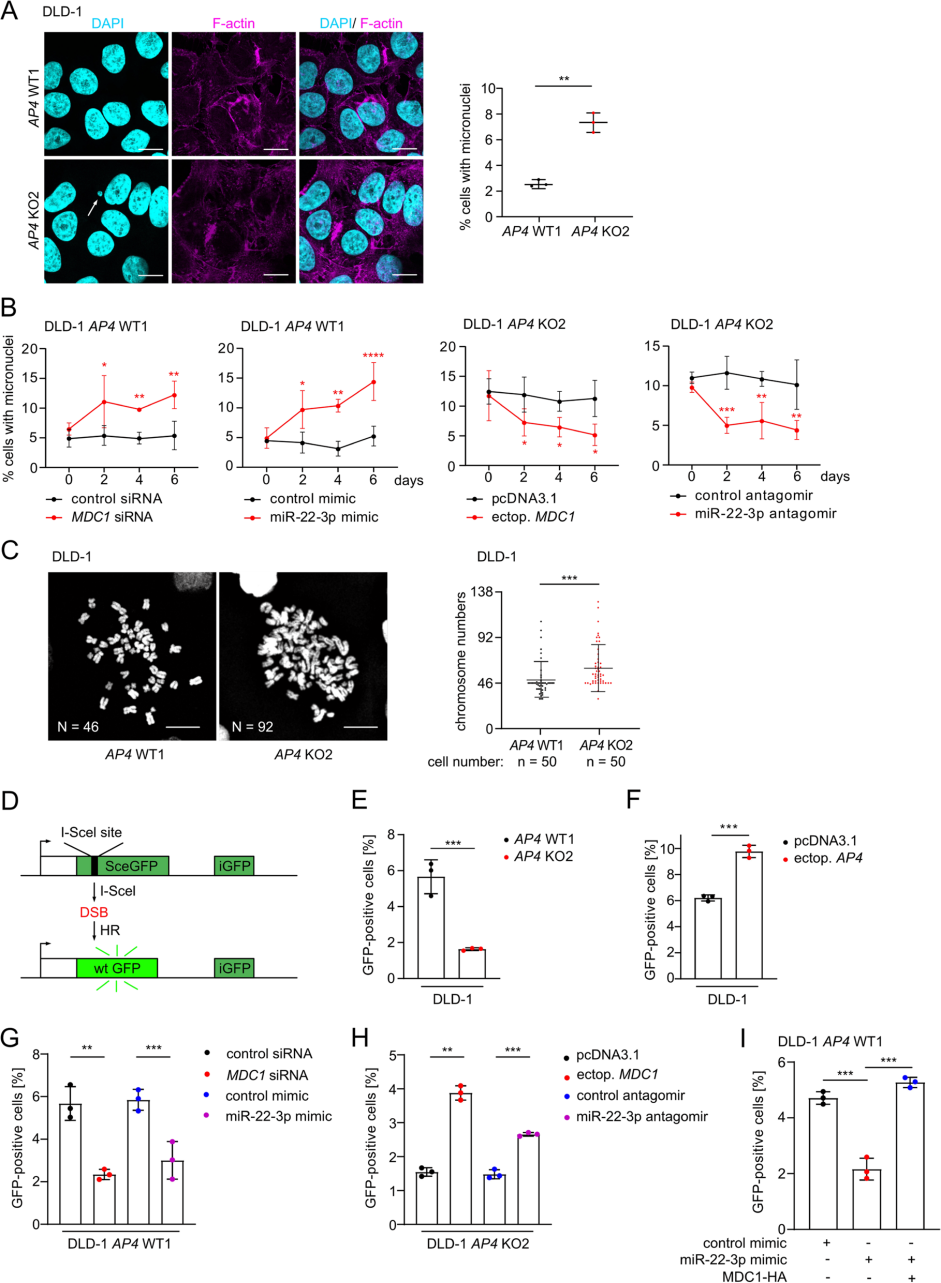
MDC1 mediates effects of AP4 on chromosomal instability. **A** Examples and quantification of micronuclei after DAPI staining. Three fields of 120 cells in total were evaluated. Scale bars: 20 μm. **B** Kinetic evaluation of micronucleus formation 48 h after transfection with the indicated oligonucleotides or vector. **C** Representative images of mitotic chromosome spreads. Quantification of 50 spreads per genotype. Scale bars: 20 μm. **D** Scheme illustrating the assay used for the fluorescence based measurement of HR-mediated DSB repair. **E** The indicated cells were co-transfected with pDR-GFP and pCBAScel plasmids. A pcDNA-mCherry plasmid was co-transfected as a control of transfection efficiency. The percentage of cells expressing GFP was measured by flow cytometry. **F**-**I** The percentage of the indicated cells expressing GFP was measured by flow cytometry 72 h after transfection of the indicated plasmids or oligonucleotides. In **A**-**B** and **E**-**I** the mean + SD (*n* = 3) and in **C** the mean + SD (*n* = 50) are provided with *: *p* < 0.05, **: *p* < 0.01, ***: *p* < 0.001, ****: *p* < 0.0001

Since DNA damage, which is not repaired before cells enter mitosis, may result in chromosomal instability (CIN) [29, 30], we determined the effect of *AP4* inactivation on CIN. Micronuclei are acentric fragments or chromosomes that failed to integrate into daughter nuclei in mitosis and can be used as a proxy for CIN [31]. Indeed, the inactivation of *AP4* in DLD-1 or SW480 cells resulted in an increased frequency of micronuclei (Fig. 8A and Fig. S6A). The frequency of micronuclei was also increased following siRNA-mediated down-regulation of *MDC1* and transfection of a miR-22-3p mimic in *AP4*-proficient DLD-1 and SW480 cells (Fig. 8B and Fig. S6B). Ectopic expression of MDC1 or miR-22-3p-specific antagomirs reduced the number of micronuclei in *AP4*-deficient DLD-1 or SW480 cells (Fig. 8B and Fig. S6B). In *AP4*-deficient DLD-1 cells the overall frequency of aberrant chromosome gains was significantly higher when compared to *AP4*-proficient DLD-1 cells (Fig. 8C). Furthermore, the aberration of chromosome numbers paralleled the formation of micronuclei in *AP4*-deficient CRC cells. In summary, the increased CIN observed in *AP4*-deficient CRC cells is, at least in part, due to a decrease of *MDC1* and an increase of miR-22-3p expression. The effects of unrepaired DNA damage on CIN occurs mainly during the G2/M transition. During this phase homologous recombination (HR) is the main pathway for repair of dsDNA breaks (DSB). Since MDC1 plays an important role in the HR [32], we asked whether AP4 regulates HR via repressing miR-22-3p and inducing MDC1. To this end, we employed a previously established HR assay [14] to evaluate HR-mediated repair of a DSB induced by the I-SceI restriction enzyme (Fig. 8D). Notably, HR was repressed in *AP4*-deficient DLD-1 and SW480 cells (Fig. 8E and Fig. S6C), whereas ectopic *AP4* enhanced HR in DLD-1 and SW480 cells (Fig. 8F and Fig. S6D). Furthermore, HR was repressed by ectopic *MDC1* siRNA or miR-22-3p mimic in *AP4*-proficient DLD-1 and SW480 cells (Fig. 8G and Fig. S6E). In addition, ectopic expression of *MDC1* or miR-22-3p antagomir restored HR in *AP4*-deficient DLD-1 and SW480 cells (Fig. 8H and Fig. S6F). Finally, a miR-22-3p-mediated decrease in HR was reversed by expression of miR-22-3p-insensitive *MDC1* in *AP4*-proficient DLD-1 and SW480 cells (Fig. 8I and Fig. S6G), implying that MDC1 is the relevant miR-22-3p target mediating the inhibitory effect of miR-22-3p on HR. Taken together, elevated AP4 expression enhances HR activity via repressing miR-22-3p and inducing MDC1 and thereby contributes to genomic integrity. The increase of CIN on *AP4*-deficient cells may therefore be a consequence of insufficient HR activity.

### AP4 modulates the sensitivity to chemotherapeutic drugs

**Fig. 9:**
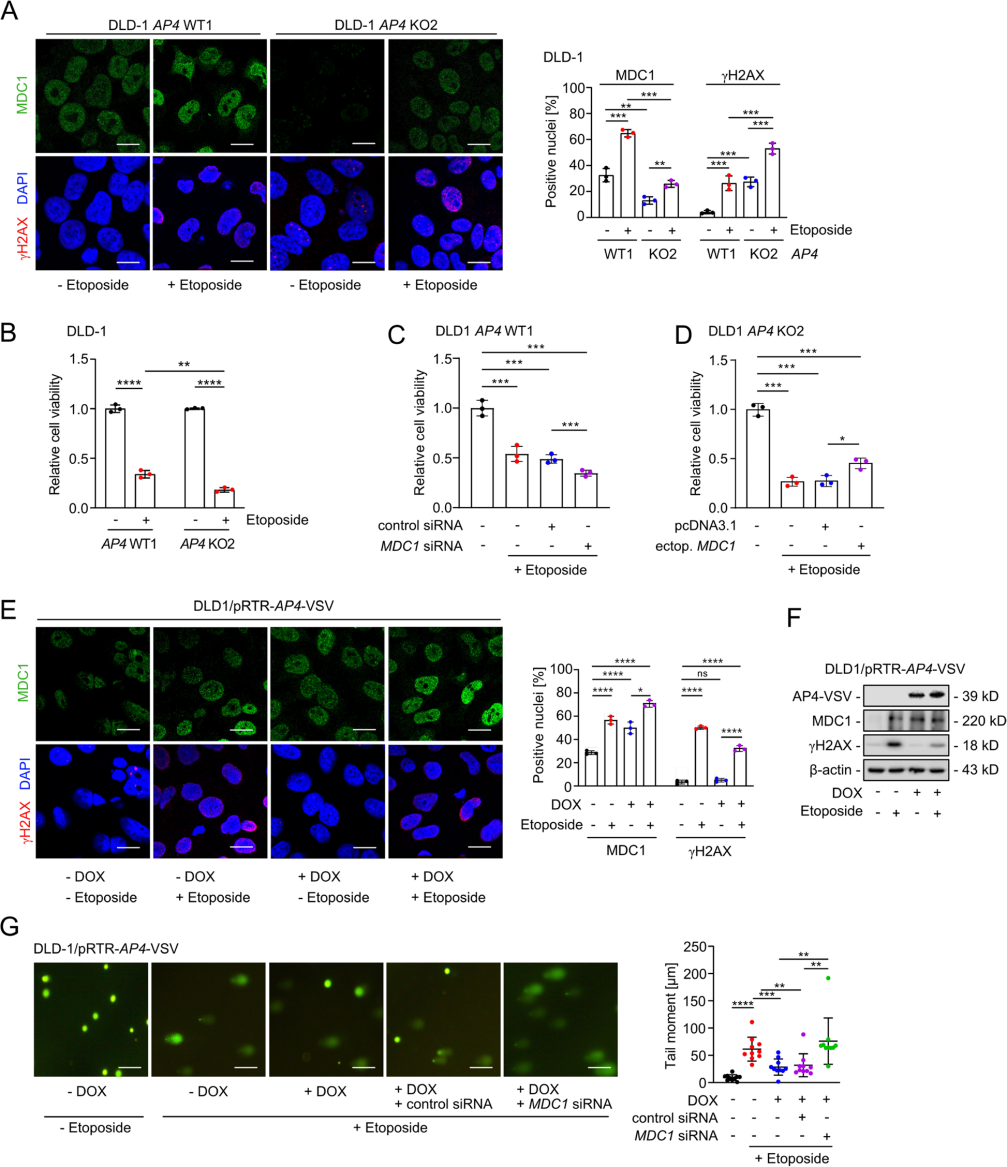
AP4 confers resistance towards Etoposide via MDC1. **A** Immunofluorescence analysis 12 h after addition of 20 μM Etoposide. Quantification of 3 fields with 120 cells in total. Scale bars: 20 μm. **B** MTT assay was performed 60 h after treatment with 20 μM Etoposide for 12 h. MTT assay of cells transfected with **C** the indicated oligonucleotides or **D** expression plasmids for 48 h and then subjected to treatment with Etoposide for 12 h. **E** Immunofluorescence analysis after activation of ectopic AP4 expression by treatment with DOX for 48 h and addition of Etoposide for 12 h. Quantification of 3 fields with 120 cells in total. Scale bars: 20 μm. **F** Western blot analysis of cells treated as in (**E**). **G** DNA damage detection by comet assay. Forty-eight hours after activation of ectopic AP4 expression by treatment with DOX, cells were transfected with the indicated oligonucleotides for 48 h and then treated with Etoposide for 12 h. For the last 60 h fresh DOX was added. Quantification of DNA tail moment by evaluation of 10 fields with 150 cells in total, Scale bars: 10 μm. The mean + SD is provided in **A-E** with (*n*=3) and in **G** with (n=10) and *:*p* < 0.05, **:*p* < 0.01, ***:*p* < 0.001, ****:*p* < 0.0001

Since AP4 enhanced the repair of DNA damage via regulation of miR-22-3p and MDC1, we asked whether AP4 decreases the sensitivity towards DNA-damage inducing chemotherapeutic drugs, such as Etoposide and 5-Fluoro-Uracil/5-FU. As shown before [33], the frequency and intensity of MDC1- and γH2AX-positive foci was elevated after treatment of CRC cells with Etoposide (Fig. 9A and Fig. S7A). In *AP4*-deficient cells more γH2AX foci were observed after Etoposide treatment when compared to *AP4*-proficient cells. In addition, *AP4* inactivation further enhanced the Etoposide-mediated reduction of cell viability (Fig. 9B and Fig. S7B), suggesting that AP4 may suppress the adverse effects of etoposide by enhancing DNA damage repair. Indeed, silencing of MDC1 enhanced the effect of Etoposide on reducing cell viability in *AP4*-proficient DLD-1 and SW480 cells (Fig. 9C and Fig. S7C). Conversely, the viability of *AP4*-deficient cells was increased by ectopic MDC1 expression after exposure to Etoposide (Fig. 9D and Fig. S7D). Therefore, the relative decrease in viability of *AP4*-deficient compared to *AP4*-proficient cells is presumably due to the increased DNA damage caused by the decreased expression of MDC1. Interestingly, ectopic AP4 expression further increased the intensity of MDC1-positive, nuclear foci in the absence and presence of Etoposide (Fig. 9E). Interestingly, the intensity and frequency of γH2AX-positive foci was decreased in DLD-1 cells ectopically expressing AP4 and exposed to Etoposide when compared to DLD-1 cells treated with Etoposide alone (Fig. 9E). Similar results were obtained by Western blot analysis (Fig. 9F). In addition, exposure to Etoposide caused an extension of the so-called “comet tails”, indicating an increase in unrepaired DNA damage (Fig. 9G). Notably, this increase was largely suppressed by ectopic AP4 expression, whereas silencing *MDC1* largely abrogated this effect of AP4. These results demonstrate that elevated AP4 expression facilitates the repair of DNA damage by inducing *MDC1.* By decreasing the amount of DNA damage elevated AP4 expression may therefore contribute to an increased viability and drug resistance of CRC cells.

**Fig. 10:**
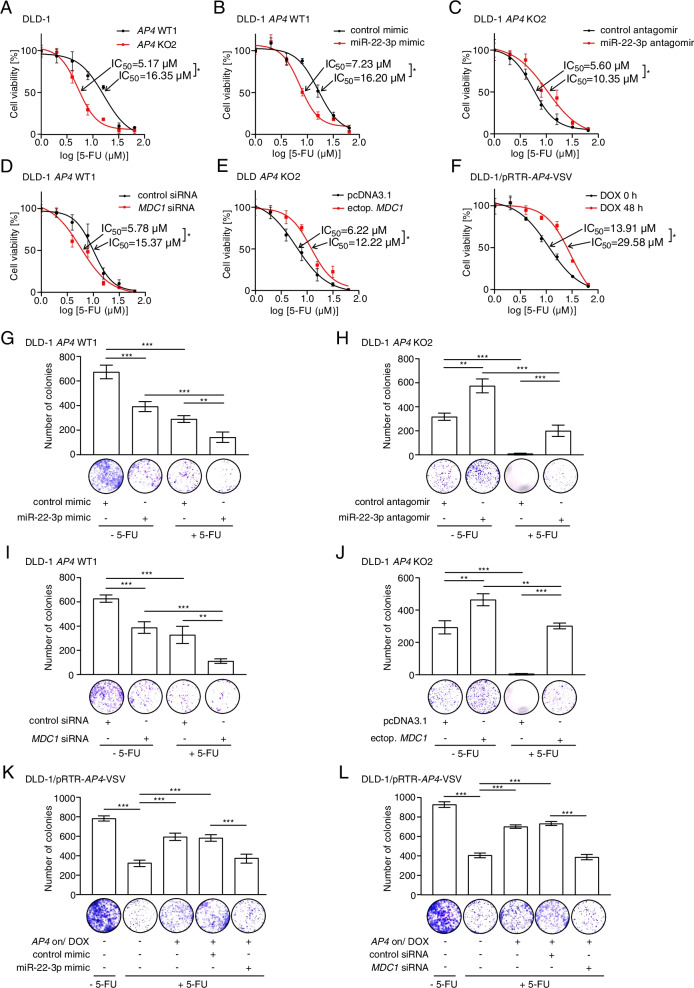
AP4 confers resistance towards 5-FU via MDC1. **A** The indicated cells were treated with increasing concentrations of 5-FU for 48 h. Then the IC_50_ was determined by an MTT assay. **B**-**E** The indicated cells were transfected with the indicated oligonucleotides for 48 h and subsequently treated with increasing concentrations of 5-FU for 48 h. Then the IC_50_ was determined by an MTT assay. **F** The DLD-1 pRTR-*AP4*-VSV cell pool was treated with DOX for 48 h and subsequently with increasing concentrations of 5-FU for 48 h. For the last 48 h fresh DOX was added. Then the IC_50_ was determined by an MTT assay. **G**-**J** Colony formation assay of the indicated cells transfected with the indicated oligonucleotides or plasmids for 48 h and then subjected to treatment with 10 μM 5-FU for 48 h and then cultured for additional 3 weeks. **K** and **L** Colony formation assay, as described in (**G**), of DLD-1 pRTR-*AP4*-VSV pools after DOX treatment for 48 h after transfection of the indicated oligonucleotides for 48 h. In panels **G**-**L**, the mean + SD is provided. *:*p* < 0.05, **:*p* < 0.01, ***:*p* < 0.001

Next, we determined whether the *AP4* status influences the sensitivity of CRC cell lines to 5-FU, a chemotherapeutic drug commonly used to treat advanced CRC. Determination of the IC_50_ dosage revealed that *AP4*-deficiency sensitizes DLD-1 cells to 5-FU when compared to *AP4*-proficient DLD-1 cells (Fig. 10A). Next, we determined whether modulation of MDC1 or miR-22-3p expression in CRC lines with varying *AP4* status affects 5-FU resistance. Indeed, ectopic expression of miR-22-3p in *AP4*-proficient DLD-1 cells reduced the tolerance to 5-FU, whereas antagomir-mediated inactivation of miR-22-3p increased the 5-FU resistance in DLD-1 *AP4* KO2 clone (Fig. 10B-C). Furthermore, silencing of MDC1 sensitized *AP4*-proficient DLD-1 cells to 5-FU (Fig. 10D). In addition, ectopic MDC1 expression enhanced 5-FU resistance in *AP4*-deficient DLD-1 cells (Fig. 10E). Furthermore, ectopic AP4 enhanced the tolerance of DLD-1 cells to 5-FU (Fig. 10F). Similar results were obtained in SW480 cells (Fig. S8A-E).

Next, we performed a colony-formation assay over a period of 3 weeks after a treatment with 5-FU for 48 h (Fig. 10G-L). In this assay, ectopic miR-22-3p expression sensitized *AP4*-proficient DLD-1 cells to 5-FU, whereas inactivation of miR-22-3p increased 5-FU resistance of *AP4*-deficient DLD-1 cells (Fig. 10G-H). When *MDC1* was silenced in *AP4*-proficient DLD-1 cells a decrease in colony numbers was observed (Fig. 10I). Conversely, ectopic *MDC1* expression increased the number of colonies of *AP4*-deficient DLD-1 cells (Fig. 10J). Similar results were also obtained in SW480 cells (Fig. S8F-I). Furthermore, ectopic *AP4* expression also increased the number of colonies of DLD-1 cells, whereas both ectopic miR-22-3p and silencing of *MDC1* abrogated the effect of AP4 (Fig. 10K-L). Taken together, these results show that elevated AP4 expression mediates 5-FU resistance in CRC cell lines in a *MDC1* and miR-22-3p dependent manner.

In addition, we analyzed the potential association between the response to chemotherapy and the expression of *c-MYC*, *AP4*, *MIR22HG* or *MDC1* in primary CRCs (Fig. S9A-D). After receiving chemotherapy, patients with CRCs displaying high expression of *c-MYC*, *AP4* or *MDC1* showed a trend towards decreased relapse free survival, when compared to patients with low expression of these genes in primary CRCs. Notably, low expression of *MIR22HG* was significantly associated with decreased relapse free survival after chemotherapy. These results suggest, that high *c-MYC*, *AP4*, *MDC1* and/or low *MIR22HG*/miR-22-3p expression in primary CRCs confer resistance to chemotherapy, whereas low expression of *c-MYC*, *AP4*, *MDC1* and/or high *MIR22HG*/miR-22-3p expression sensitize to chemotherapy.

## Discussion

Here we identified a new regulatory connection that explains how AP4 preserves genomic integrity in the context of c-MYC activation (see also graphical abstract). Our results demonstrate that *AP4* suppresses DNA damage, which occurs spontaneously or at an increased rate after c-MYC activation in CRC cells, by promoting the expression of *MDC1*. By forming a coherent feed-forward loop, in which AP4 directly induces *MDC1* and represses miR-22-3p, a known inhibitor of MDC1, AP4 can exert a tight and robust regulation of MDC1 expression. Notably, MDC1 is a central effector in the DNA damage response/DDR as it serves as a molecular platform for many DNA repair proteins at the site of DNA damage and facilitates the amplification of the initial γH2AX signal [22–24]. By regulating the abundance of MDC1 protein, AP4 is therefore able to fine-tune the cellular response to DNA damage. In support of this model, ectopic AP4 suppressed DNA damage induced by Etoposide and 5-FU in a manner dependent on MDC1 and miR-22-3p. Thereby, AP4 may contribute to resistance of CRC cells towards DNA damaging substances. Since we obtained similar results in MSI/microsatellite instable (DLD-1) and MSS/microsatellite stable (SW480) CRC cell lines, our results are presumably relevant for the majority of CRCs, as these fall into either of these categories. As both cell lines harbor mutant *p53* alleles, the effects of AP4 on the DDR are independent of wild-type *p53*. Since *AP4*, *c-MYC* and *MDC1* expression are concomitantly elevated in primary CRCs along with down-regulation of *MIR22HG*, these findings are presumably of clinical relevance.

Our results imply that patients with CRCs that display low expression of c-*MYC*, *AP4* and *MDC1,* as well as elevated miR-22-3p levels should respond better to chemotherapy than patients with CRCs that exhibit high c-*MYC*, *AP4*, *MDC1* and low miR-22-3p expression. Indeed, we detected an association between poor response to chemotherapy and elevated c-*MYC*, *AP4* and *MDC1* expression, as well as low *MIR22HG* expression in a cohort of CRC patients. The mechanism underlying this effect is likely to be the increased DNA repair capacity of cells with elevated AP4 (and therefore high MDC1 levels) detected in this study. Therefore, further studies to validate the use of AP4 and MDC1 as predictive markers are warranted.

Furthermore, our results indicate that inhibition of AP4 sensitizes cancer cells to DNA damaging agents used for chemotherapy. In the future inhibition of AP4 function may be achieved by inhibition of AP4 homo-dimerization using small-drugs or synthetic peptides. Similar approaches have been used to interfere with c-MYC/MAX hetero-dimerization [34]. Alternatively, targeted degradation approaches, such as PROTAC/PROteolysis TArgeting Chimeras, may be used to down-regulate AP4 protein levels [35].

Interestingly, decreased expression of *MIR22HG* in primary CRCs is associated with poor survival of CRC patients [36]. In addition, elevated expression of AP4 protein showed a significant correlation with distant metastasis and advanced tumor grade in CRC patients [19]. Therefore, the detection of *MIR22HG* and/or AP4 expression may also have prognostic value for CRC.

MiR-22-3p has been characterized as a senescence-associated microRNA that functions by directly targeting and suppressing *CDK6*, *SIRT1*, *Sp1* and *MDC1* [20, 21]. Expression of an MDC1 variant insensitive to miR-22-3p alleviated DNA damage and senescence caused by miR-22-3p. Therefore, other miR-22-3p targets besides MDC1 are presumably not relevant in the context of c-MYC/AP4 activation and the senescence observed in *AP4*-deficient cells was largely due to elevated expression of miR-22-3p and the resulting decreased expression of *MDC1*.

Previous studies have shown that *c-MYC* induces G_1_/S transition and DNA replication by activating CDKs and also by directly interacting with replication associated proteins [37, 38]. In tumor cells c-MYC expression is often deregulated and the resulting unscheduled DNA replication causes DNA damage and genomic instability [39, 40]. Here, *MDC1* expression was increased to a larger extent after *c-MYC* activation in *AP4*-proficient than in *AP4*-deficient CRC cells, which inversely correlated with the amount of DNA damage. Therefore, the AP4/*MDC1* axis protects cells from *c-MYC*-induced DNA damage. In addition, repression of *MDC1* by siRNA and miR-22-3p resulted in an increased frequency of micronuclei in *AP4*-proficient cells, indicating that the AP4-mediated repression of miR-22-3p and the resulting de-repression of *MDC1* is critical for maintaining chromosomal stability. Taken together, AP4-induced MDC1 therefore limits the extent of DNA damage that cells encounter after activation of c-MYC and thereby allows the proliferation of cancer cells with deregulated c-MYC expression. Interestingly, *MDC1* is also necessary for the intra-S-phase and the G_2_/M DNA damage checkpoints [23, 24]. Furthermore, repressing MDC1 induces apoptotic cell death following DNA damage caused by ionizing radiation [41].

In primary CRCs expression of c-*MYC*, *AP4* and *MDC1* was consistently up-regulated. Interestingly, elevated MDC1 levels have also been reported in cervical, laryngeal squamous and nasopharyngal carcinomas, which are associated with viral infections and expression of viral proteins, and therefore high levels of DNA damage [42–44]. Since up-regulation of c-*MYC* is a hallmark of CRCs [45], it is therefore tempting to speculate that CRCs harbor comparatively high levels of DNA damage due to elevated expression of c-MYC, which results in a requirement of MDC1 up-regulation for tumor initiation and progression. A further plausible source of DNA damage during CRC initiation and progression are presumably the products of certain bacterial strains, such as Colibactin produced by *pks* + *E. coli*, which contribute to transformation of colon epithelial cells [46].

5-FU has become the mainstay of systemic treatment of CRC since the 1990s. However, nearly 50% of patients diagnosed with metastatic CRC have a low five-year survival rate of 12% due to resistance towards 5-FU-based chemotherapy [47]. 5-FU metabolites are incorporated into RNA and DNA [48], and are subject to base excision repair (BER) or mismatch repair (MMR). DSBs generated during the repair undergo homologous recombination (HR) [49]. Accordingly, impaired HR sensitizes cancer cells to 5-FU treatment and MMR-deficient CRCs display better clinical outcomes after chemotherapy involving 5-FU [50, 51]. Interestingly, inactivation of MDC1 impaired MMR and HR [52, 53]. Here we observed, that AP4 induces HR in CRC cell lines. In addition, ectopic MDC1 expression or inhibition of miR-22-3p restored the capacity for HR in *AP4*-deficient CRC cells. Therefore, elevated AP4 expression promotes chemo-resistance by enhancing HR via directly and indirectly increasing the levels of MDC1. Our observation that silencing of *MDC1* sensitizes *AP4*-proficient cells to 5-FU treatment is in line with previous studies in which *MDC1* inactivation resulted in increased radio-sensitivity and sensitivity to DNA-damaging chemotherapeutics [44, 54, 55]. In addition, we found that *AP4* confers 5-FU resistance via up-regulating *MDC1* and thereby enhancing the repair of 5-FU-induced DNA damage. Since it has been shown that activation of *c-MYC* also contributes to chemotherapy resistance in CRC [56], the c-*MYC*/*AP4*/miR-22-3p/*MDC1* feed-forward loop characterized here is an attractive, new molecular mechanism, which explains chemotherapy resistance in CRC with elevated *c-MYC* expression. As the majority of CRCs show elevated c-MYC and AP4 expression, interference with the c-MYC/AP4/miR-22-3p/MDC1 axis represents an attractive approach to sensitize CRCs to chemotherapies.

## Conclusions

In summary, AP4, miR-22-3p and *MDC1* form a coherent, regulatory feed-forward loop to promote DNA repair, which suppresses DNA damage, senescence and CIN, and contributes to 5-FU resistance. These findings explain how elevated AP4 expression contributes to initiation, maintenance, progression and chemo-resistance of colorectal cancer after c-MYC activation.

## Supplementary Information

**Additional file 1.**

## Data Availability

All data generated or analyzed during this study are included in this published article and its supplementary information files.

## Abbreviations

AP4: transcription factor AP-4
*APC*: *Adenomatous polyposis coli*
CCLE: Cancer Cell Line Encyclopedia
CIN: Chromosomal instability
CMS: consensus molecular subtype
COAD: colon adenocarcinoma
CRC: colorectal cancer
CRIS: CRC intrinsic subtypes
DOX: Doxycycline
HR: Homologous recombination
5-FU: 5-Fluorouracil
MDC1: Mediator of DNA damage Checkpoint 1
Min: Multiple intestinal neoplasia
MTT: 3-(4,5-dimethylthiazol-2-yl)-2,5-diphenyltetrazolium bromide
*MIR22HG*: MIR22 host gene
PDX: patient-derived xenografts
qChIP: quantitative chromatin immunoprecipitation
TCGA: The Cancer Genome Atlas
TSS: transcriptional start site
UTR: Untranslated region
