# Supplementary material for: AP4 suppresses DNA damage, chromosomal instability and senescence via inducing MDC1/Mediator of DNA damage Checkpoint 1 and repressing MIR22HG/miR-22-3p
Source: Mol Cancer. 2022 May 27;21:120. doi: 10.1186/s12943-022-01581-1 (PMC9137087; doi:10.1186/s12943-022-01581-1)
Supplement: Supplementary file 1 — Additional file 1. [file 12943_2022_1581_MOESM1_ESM.pdf]

Supplementary information

AP4 suppresses DNA damage, chromosomal instability and senescence via inducing *MDC1/Mediator of DNA damage Checkpoint 1* and repressing *MIR22HG*/miR-22-3p

Chou *et al*.

List of contents:

Additional file 1: Fig. S1: *AP4* inactivation induces senescence and apoptosis in CRC cells.

Additional file 2: Fig. S2: *AP4* inactivation induces DNA damage in CRC cells.

Additional file 3: Fig. S3: AP4 directly represses *MIR22HG.*

Additional file 4: Fig. S4: *MDC1* is directly and indirectly regulated by AP4.

Additional file 5: Fig. S5: MDC1 mediates effects of *AP4* on DNA damage and senescence.

Additional file 6: Fig. S6: MDC1 mediates effects of *AP4* on chromosomal instability and HR.

Additional file 7: Fig. S7: AP4 confers resistance towards Etoposide via MDC1.

Additional file 8: Fig. S8: AP4 confers resistance towards 5-FU via MDC1.

Additional file 9: Fig. S9: Kaplan–Meier analysis of the association between relapse free survival and the mRNA expression.

Additional file 10: Fig. S10: Original blots.

Additional file 11: Table S1: Sequence information for miR-22-3p mimic and control mimic.

Additional file 12: Table S2: Sequence information for guide RNAs used for *AP4* deletion.

Additional file 13: Table S3: List of Antibodies.

Additional file 14: Table S4: Oligonucleotides used for qPCR.

Additional file 15: Table S5: Oligonucleotides used for qChIP.

Additional file 16: Table S6: Oligonucleotides used for reporter plasmids.

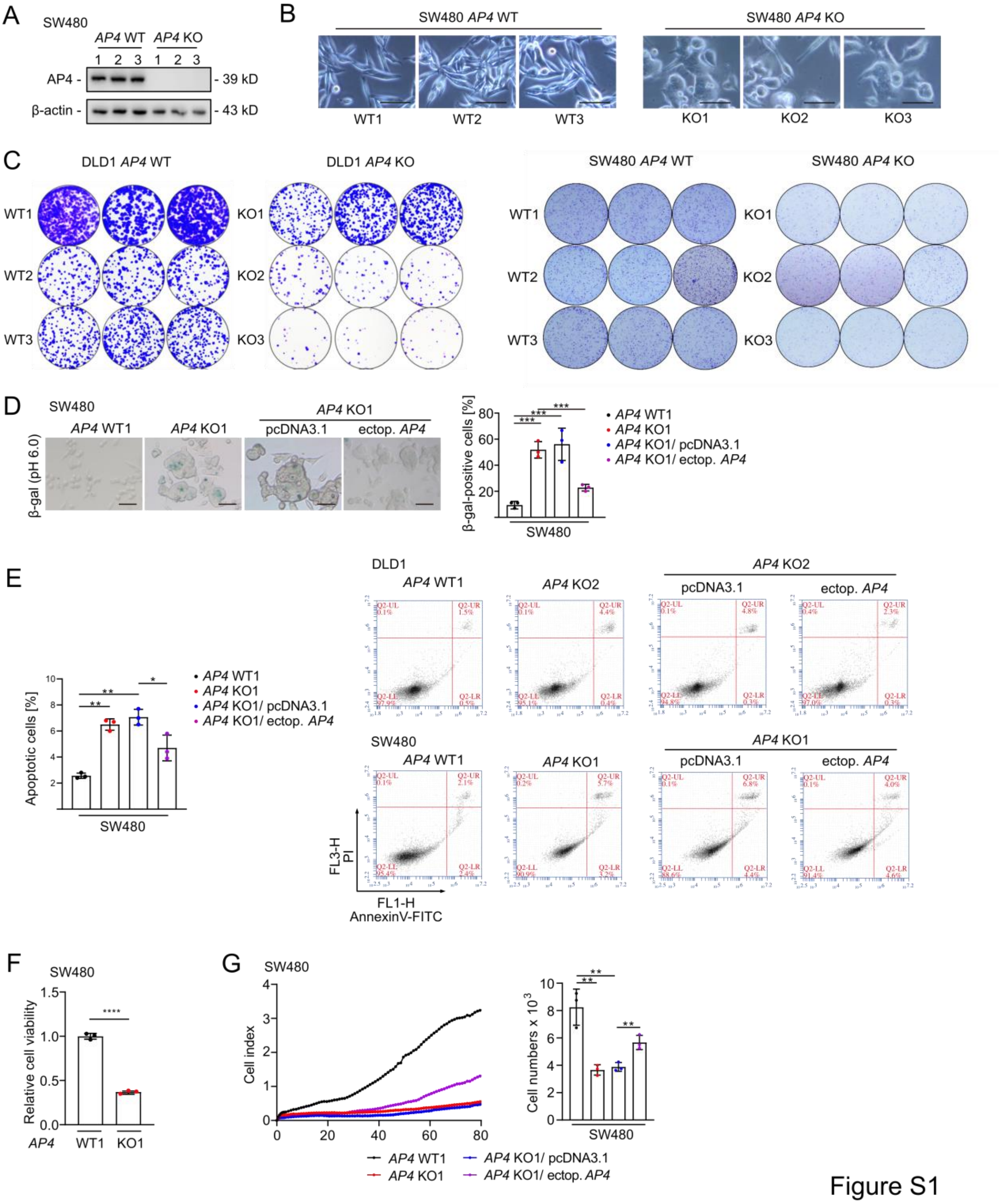

Fig. S1: *AP4* inactivation induces senescence and apoptosis in CRC cells. A AP4 detection by Western blot analysis. β-actin served as a loading control. **B** Phase contrast images of the indicated cell lines. Scale bars: 50 μm. **C** Colony formation assays of *AP4* WT and KO single cell clones obtained from DLD-1 and SW480 cells. Three independent clones were analyzed. **D** Detection of senescent cells using pH6 β-gal staining after 48 hours of transfection. Quantification of a total of 120 cells in 3 fields. Scale bars: 100 μm. **E** Quantification of apoptotic cells by Annexin V / PI detection 48 hours after transfection in the indicated cells (left panel). The flow charts of DLD-1 and SW480 cells with indicated transfections are presented in the right panel.

F MTT assay results of indicated cell lines 72 hours after seeding into a 96-well plate. **G** Proliferation was determined by impedance measurement. In panels **D-G** the mean ± SD (n = 3) is provided. *: $p < 0.05$, **: $p < 0.01$, ***: $p < 0.001$, ****: $p < 0.0001$.

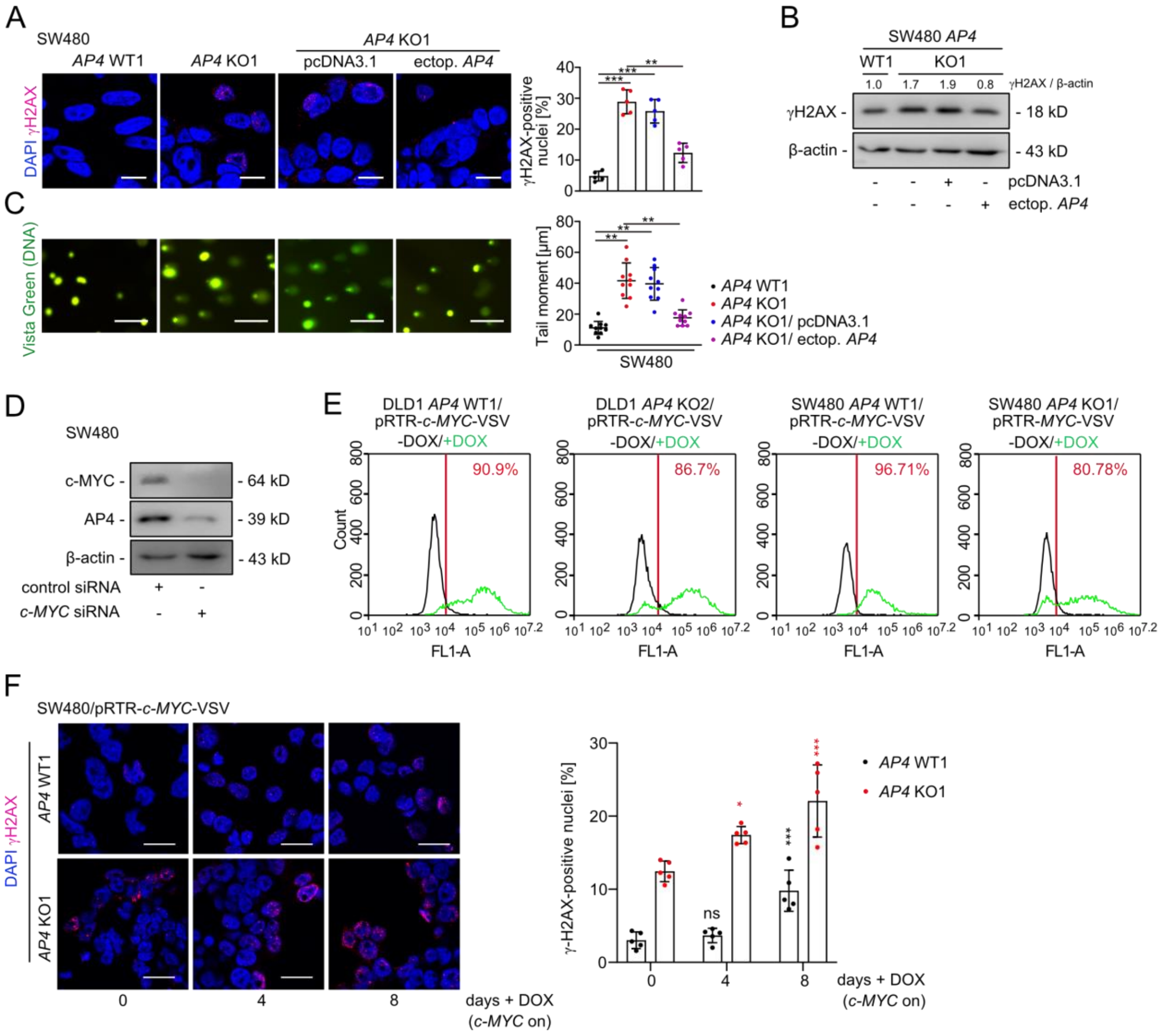

Figure S2

Fig. S2: ***AP4*** **inactivation induces DNA damage in CRC cells. A** Detection of γH2AX foci. Quantification of a total of 100 cells in 5 fields. Scale bars: 20 µm. **B** Western blot analysis of γH2AX 48 hours after transfection in the indicated cells. **C** Comet assay and quantification of DNA tail moment of a total of 150 cells in 10 fields. Scale bars: 20 µm. **D** Western blot analysis 48 hours after transfection of c-*MYC*–specific siRNA. **E** FACS analysis of DLD-1/SW480 *AP4* WT/KO pRTR-c-*MYC*-VSV pools. Pools with more than 80% GFP-positive cells were selected. **F** γH2AX detection after c-*MYC* induction by DOX for indicated durations. Quantification of a total of 100 cells in 5 fields. Scale bars: 20 µm. In panels **A** the mean ± SD (n = 5)**, C** the mean ± SD (n = 10) and **F** the mean ± SD (n = 5) are provided. *: $p < 0.05$, **: $p < 0.01$, ***: $p < 0.001$.

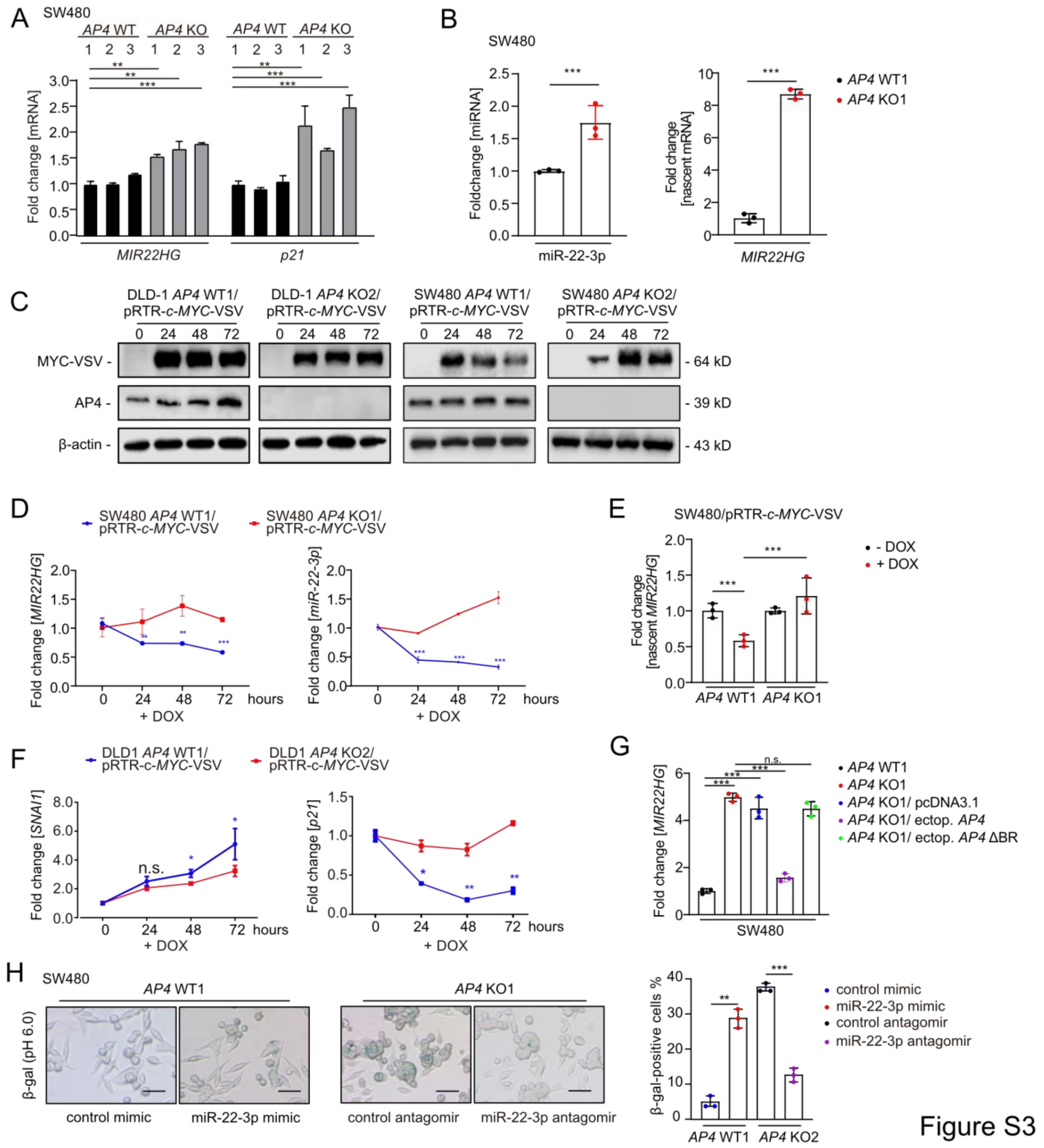

Fig. S3: AP4 directly represses *MIR22HG.* **A** qPCR analysis of *MIR22HG* and *p21* in the indicated clones of SW480 cells. **B** qPCR analysis of miR-22-3p expression (left panel) and nascent *MIR22HG* mRNA (right panel) in the indicated cells. **C** Western blot analysis of MYC-VSV and AP4 expression after c-*MYC* induction by DOX for indicated durations. **D** qPCR analysis of *MIR22HG* (left panel) and miR-22-3p (right panel) after c-MYC induction by DOX for indicated durations. **E** qPCR analysis of nascent *MIR22HG* mRNA 48 hours after c-*MYC* induction by DOX. **F** qPCR analysis of *SNAI1* (left panel) and *p21* (right panel) after c-*MYC* induction by DOX for indicated durations. **G** qPCR analysis of *MIR22HG* 48 hours after transfection. **H** β-gal detection at pH 6 48 hours after transfection. Quantification of a total of 120 cells in 3 fields. Scale bars: 50 µm. In panels **A-B**, **D-H** the mean ± SD (n = 3) is provided with *: $p < 0.05$, **: $p < 0.01$, ***: $p < 0.001$.

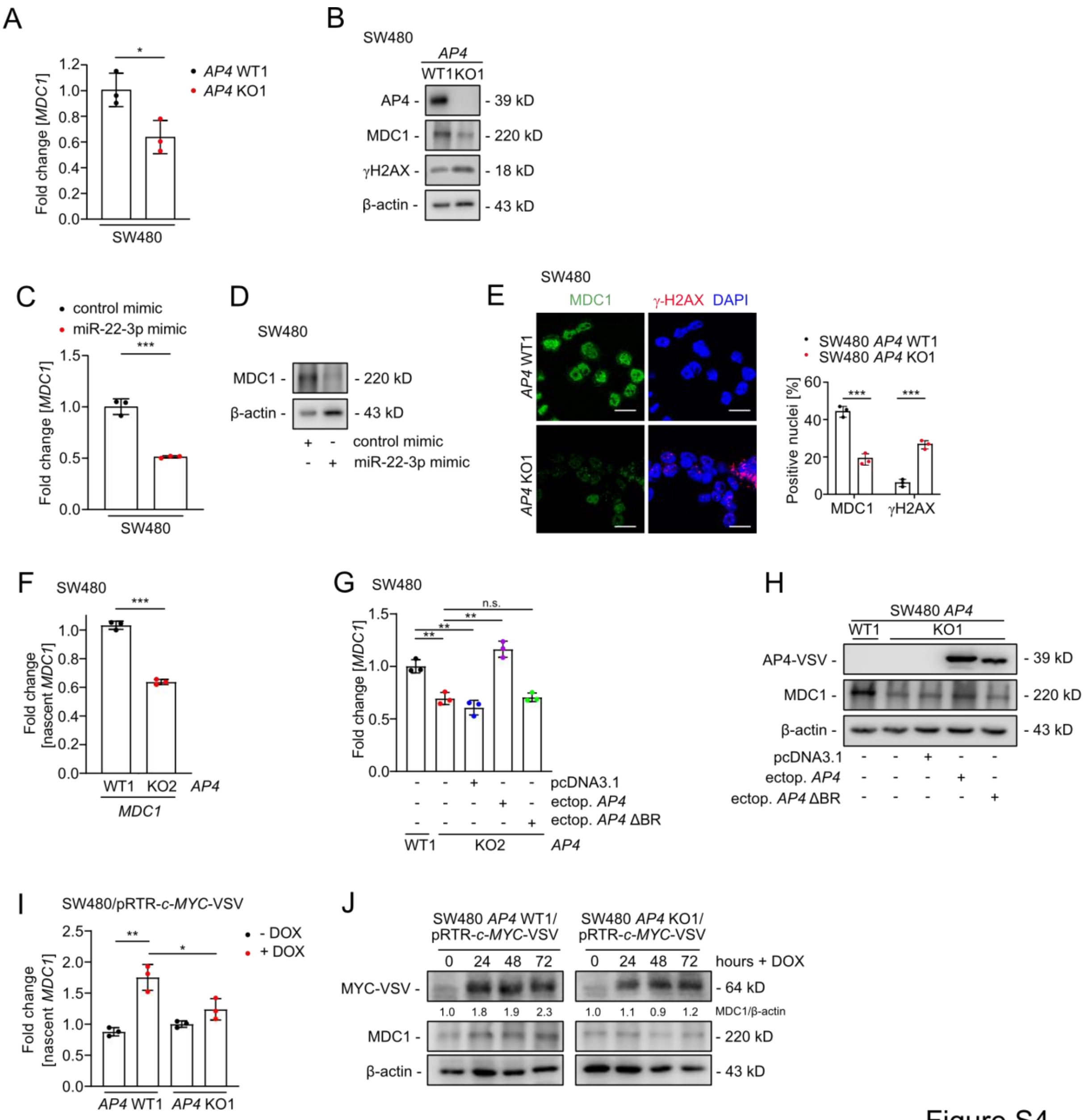

Figure S4

Fig. S4: *MDC1* is directly and indirectly regulated by AP4. A qPCR analysis of *MDC1* expression. **B** Western blot analysis. **C** qPCR analysis 48 hours after transfection. **D** Western blot analysis 48 hours after transfection. **E** MDC1 foci detection in untreated cells. Quantification of a total of 120 cells in 3 fields. Scale bars: 20 µm. **F** qPCR analysis of nascent *MDC1* mRNA in the indicated cell lines. **G** qPCR analysis of *MDC1* 48 hours after transfections. **H** Western blot analysis 48 hours after transfection. **I** qPCR analysis of nascent *MDC1* mRNA 48 hours after c-MYC induction by DOX. **J** Western blot analysis after induction of c-MYC by DOX for the indicated periods. In panels **A**, **C**, **E-G** and **I** the mean ± SD (n = 3) is provided with *: $p < 0.05$, **: $p < 0.01$, ***: $p < 0.001$.

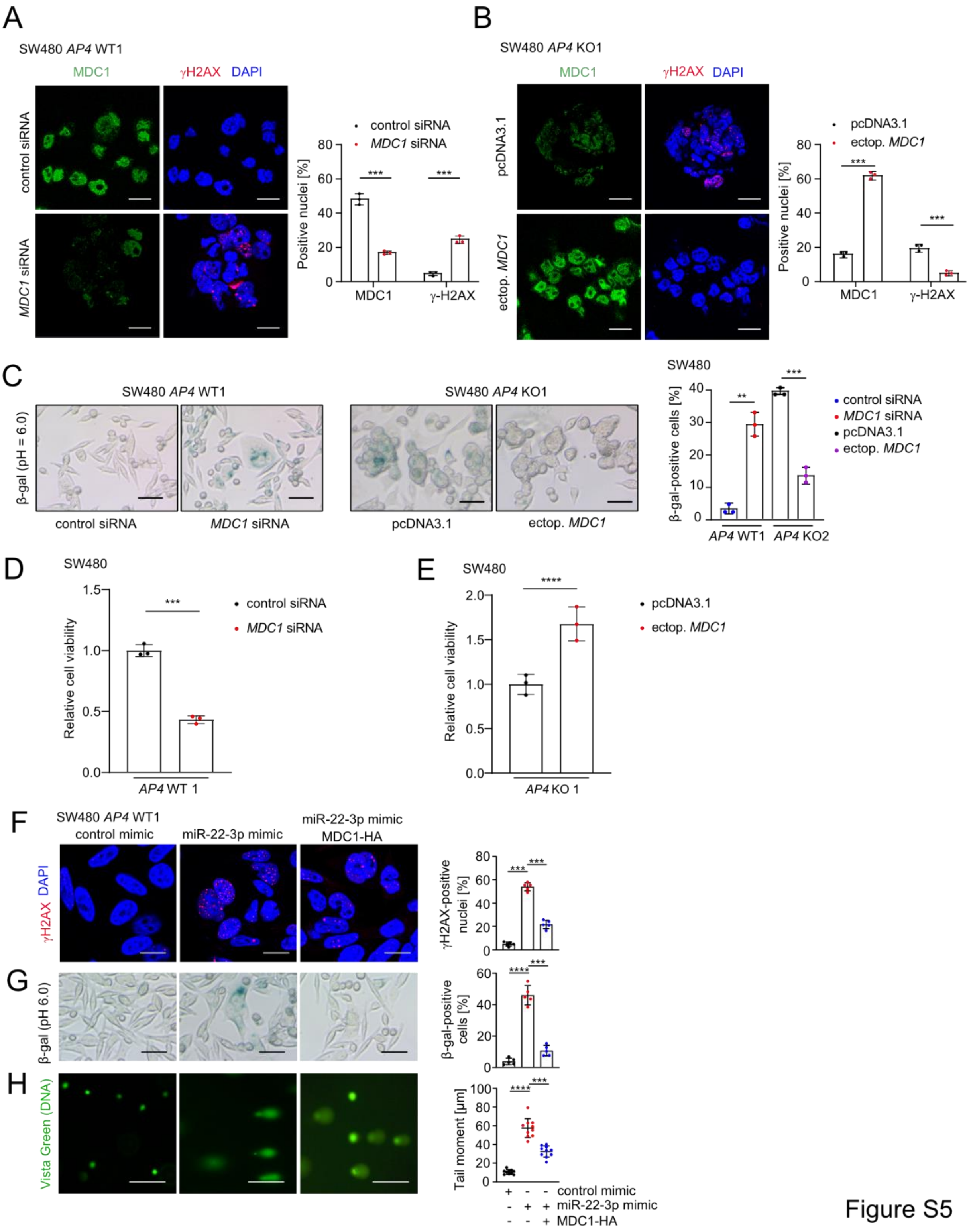

Fig. S5: MDC1 mediates effects of AP4 on DNA damage and senescence. A Detection of MDC1 and γH2AX foci by immunocytochemistry 48 hours after silencing *MDC1*. Scale bars: 20 µm. Quantification of a total of 120 cells in 3 fields. **B** MDC1 and γH2AX foci were detected by immunocytochemistry 48 hours after ectopic expression of MDC1. Scale bars: 20 µm. Quantification of a total of 120 cells in 3 fields. **C** β-gal staining 48 hours after silencing or ectopic expression of MDC1, respectively.

Quantification of a total of 120 cells in 3 fields. Scale bars: 50 µm. **D** MTT assay results 48 hours after silencing *MDC1*. **E** MTT assay results 48 hours after ectopic expressing MDC1. Detection of **F** γH2AX foci by immunocytochemistry, **G** β-gal staining and **H** comet assay in DLD-1 *AP4* WT1 cells 48 hours after transfection. Quantification of DNA tail moment of a total of 150 cells in 10 fields. In panel **A-E**, the mean ± SD (n = 3), in panel **F-G**, the mean ± SD (n = 5) and in panel **H**, the mean ± SD (n = 10) are provided with **: $p < 0.01$, ***: $p < 0.001$, ****: $p < 0.0001$.

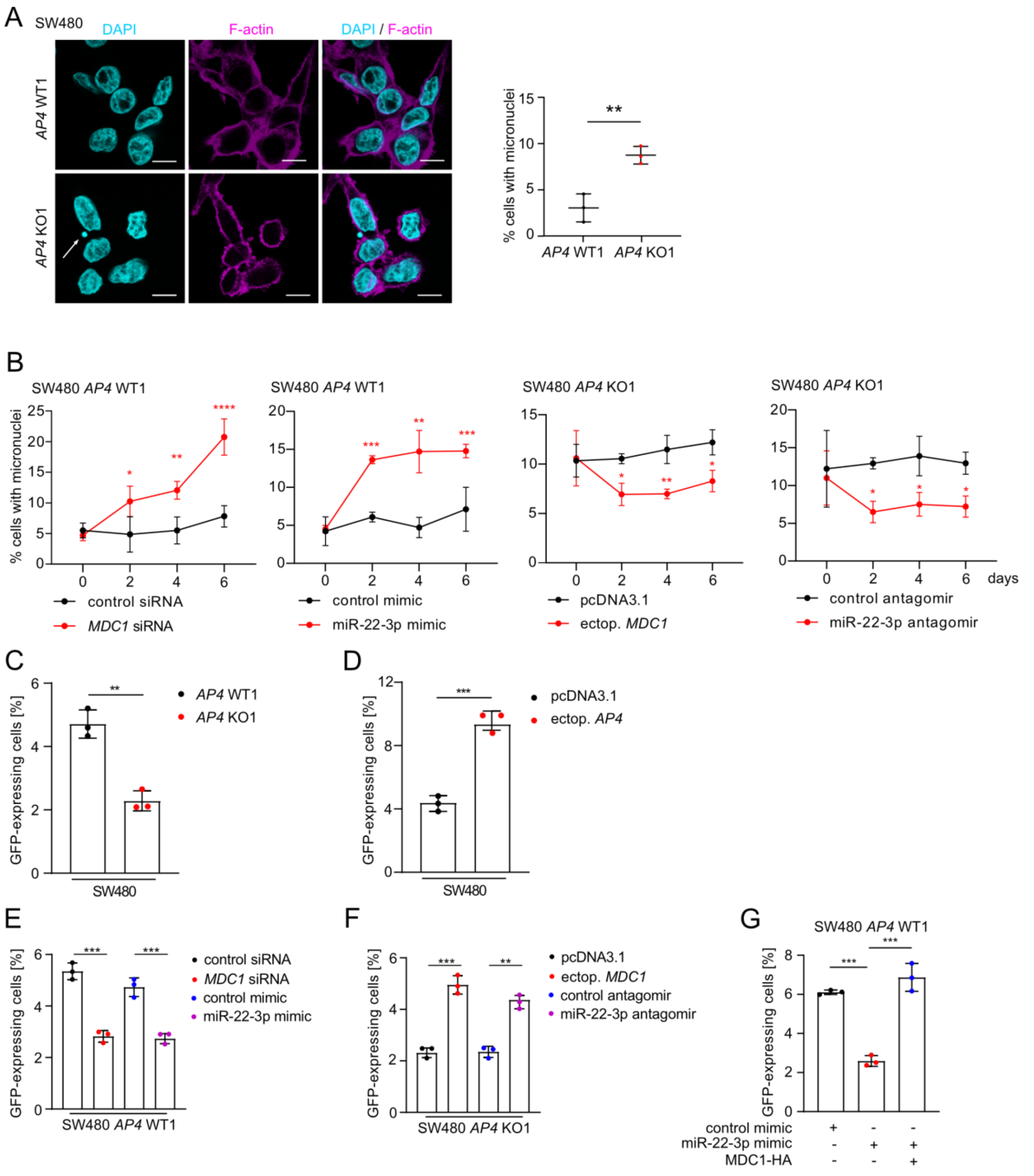

Figure S6

Fig. S6: MDC1 mediates effects of AP4 on chromosomal instability and HR. A Examples and quantification of micronuclei after DAPI staining. Quantification of a total of 50 cells in 3 fields. Scale bars: 20 µm. **B** Kinetic evaluation of micronucleus formation 48 hours after transfection with the indicated oligonucleotides or vectors. **C** The indicated cells were co-transfected with pDR-GFP, pCBAScel plasmids. A pcDNA-mCherry plasmid was also co-transfected as a control of transfection efficiency. The percentage of cells expressing GFP among mCherry-positive cells was measured by flow cytometry 72 hours after transfection. **D-G** The percentage of the indicated cells expressing GFP was measured by flow cytometry after 72 hours of transfections with the indicated plasmids or oligonucleotides. In panels **A-G,** the mean + SD (n = 3) is provided with *: $p < 0.05$, **: $p < 0.01$, ***: $p < 0.001$.

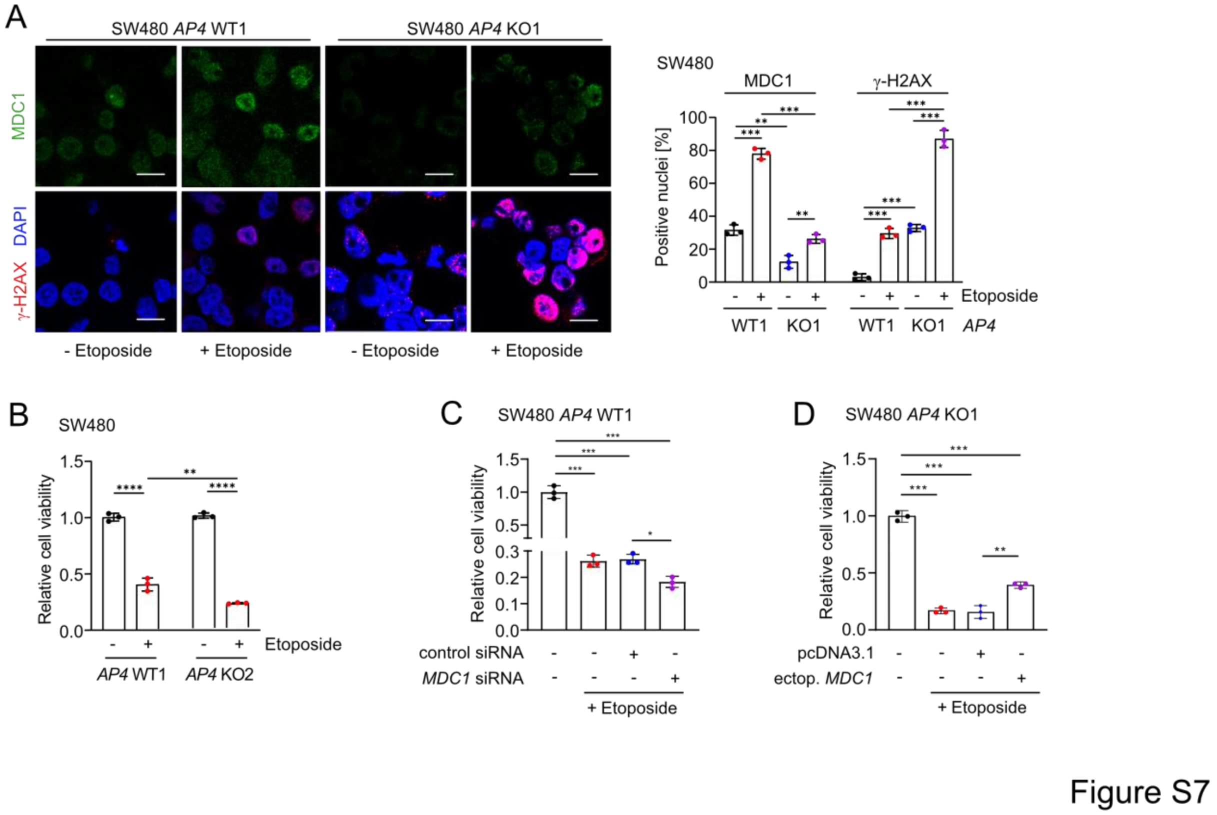

Fig. S7: AP4 confers resistance towards Etoposide via MDC1. A Immunocytochemistry detection of MDC1 and γH2AX foci 12 hours after addition of 20 μM Etoposide. Quantification of a total of 120 cells in 3 fields. Scale bars: 50 μm. **B** MTT assay results 12 hours after 20 μM Etoposide treatment. MTT assay results of cells transfected with **C** the indicated oligonucleotides or **D** expression plasmids for 48 hours and then subjected to treatment with Etoposide for another 12 hours. In panels **A-D** the mean ± SD (n = 3) is provided with *: $p < 0.05$, **: $p < 0.01$, ***: $p < 0.001$.

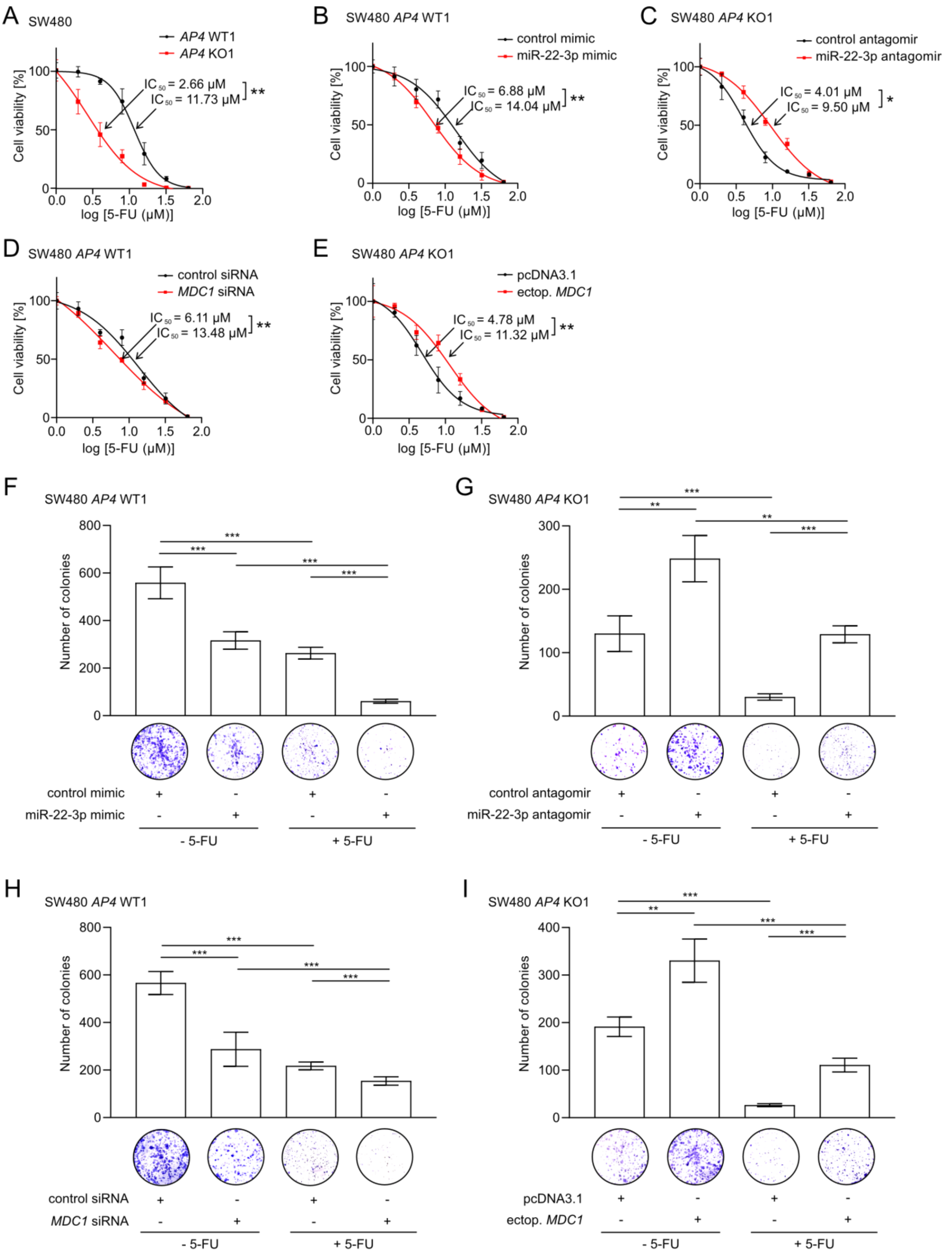

Figure S8

Fig. S8: AP4 confers resistance towards 5-FU via MDC1. A The indicated cells were treated with increasing concentrations of 5-FU for 48 hours. Then the IC_{50} was determined by an MTT assay. **B-E** The indicated cells were transfected with indicated oligonucleotides for 48 hours and subsequently treated with increasing concentrations

of 5-FU for another 48 hours. After treatments, the IC_{50} was determined by an MTT assay. **F-I** Colony formation assay of the indicated cells transfected with the indicated oligonucleotides or plasmids for 48 hours and then subjected to treatment with 10 µM 5-FU for another 48 hours. For the last 48 hours fresh DOX was added. After treatments, cells were cultured for 3 weeks. In panels **A-I**, the mean $\pm$ SD (n = 3) is provided with *: $p < 0.05$, **: $p < 0.01$, ***: $p < 0.001$.

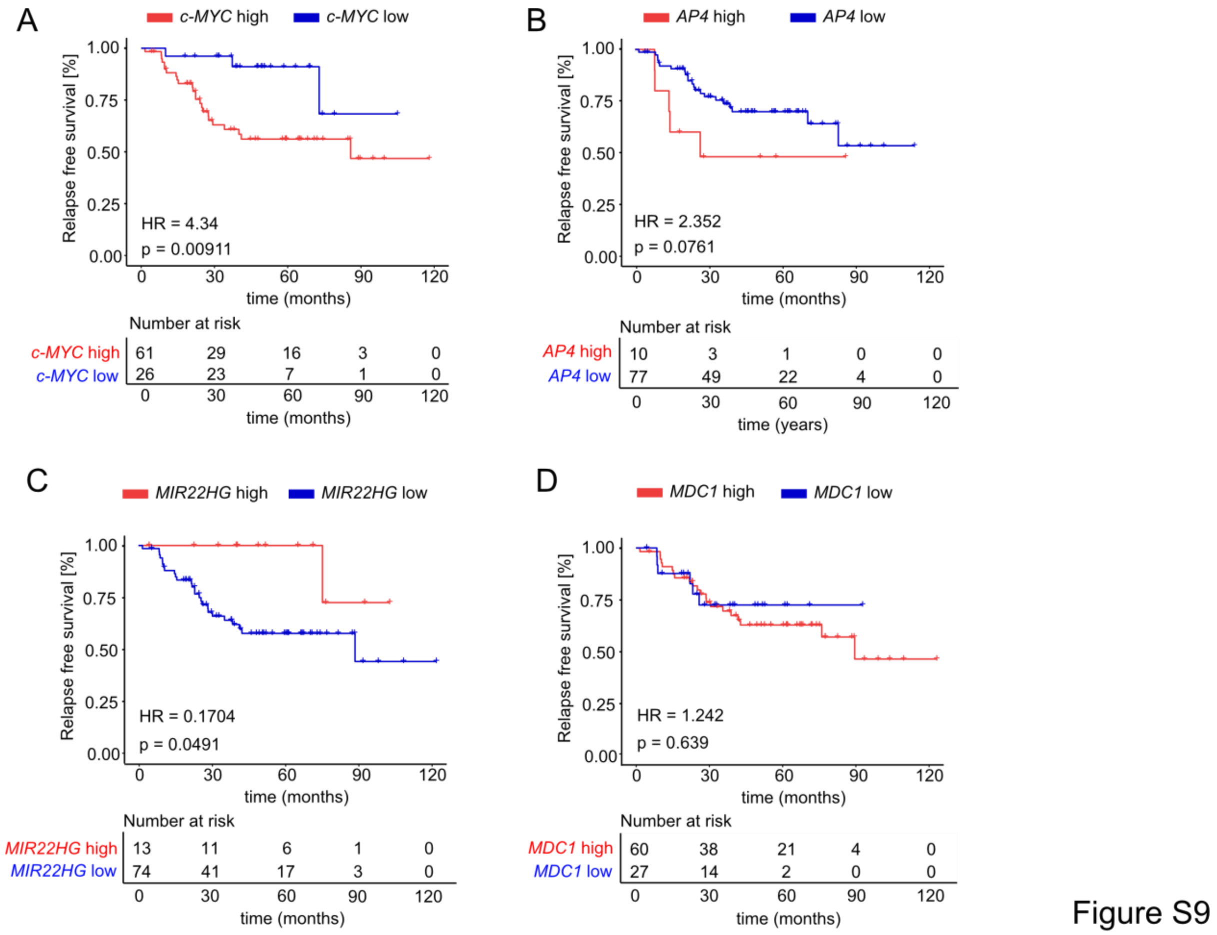

Fig. S9: Kaplan–Meier analysis of the association between relapse free survival and c-*MYC, AP4, MIR22HG* and *MDC1* mRNA expression. Analysis of **A** c-*MYC*, **B** *AP4*, **C** *MIR22HG* and **D** *MDC1* in patients that received chemotherapy (n = 87) represented in the GSE14333 dataset. The significance was calculated with the log-rank test. Below the graphs, the number of patients at risk with high or low expression of the indicated mRNA at the respective time point is provided. HR, hazard ratio.

Fig. S10: Original blots.

Uncropped gel for Figure 1B and S1A

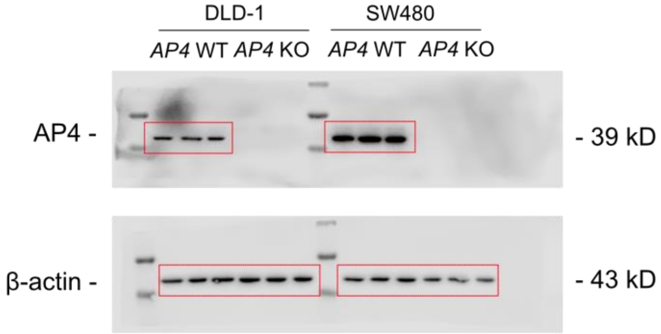

Uncropped gel for Figure 1I and S2B

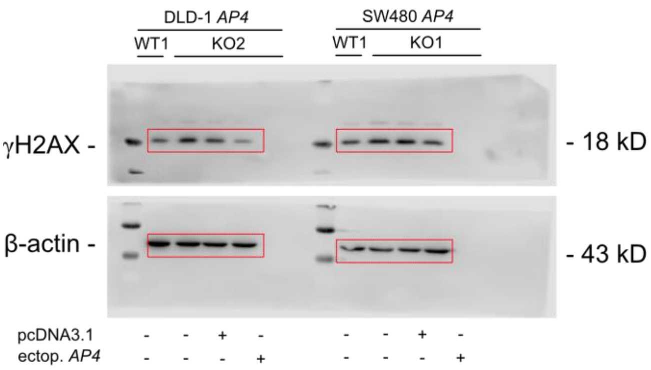

Uncropped gel for Figure 1K and S2D

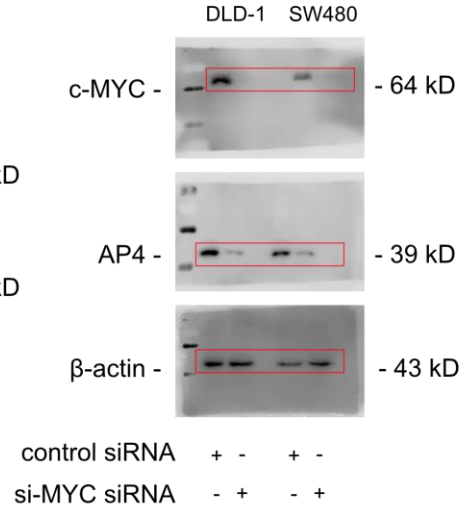

uncropped gel of Fig 3B and S4B

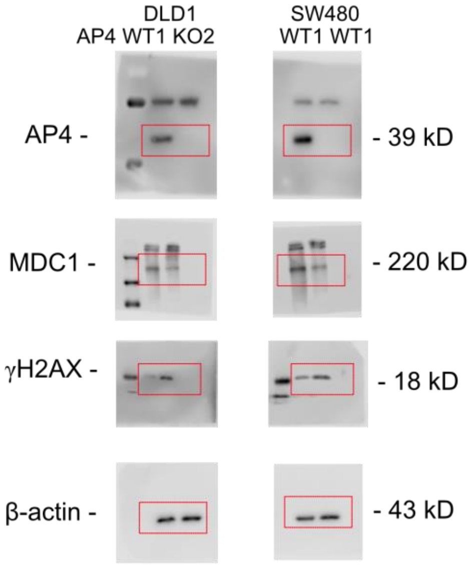

uncropped gel of Fig 3E and S4D

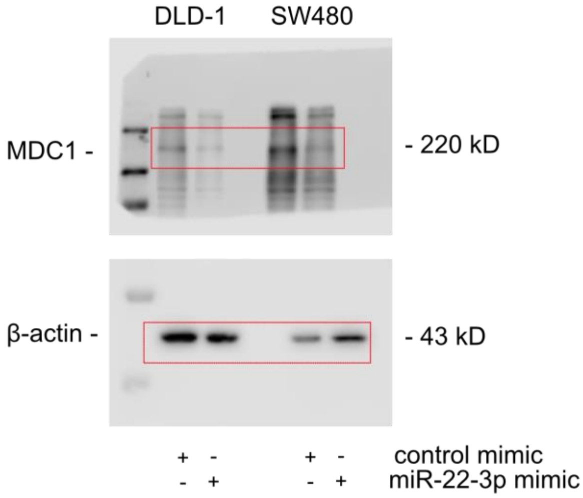

uncropped gel of Fig 4E and S4H

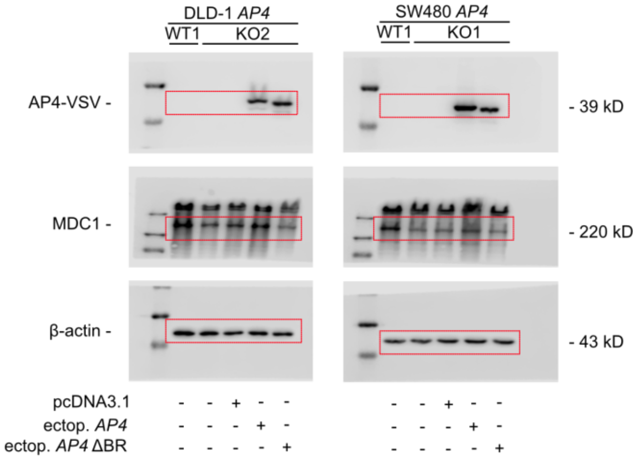

uncropped gel of Fig 4F

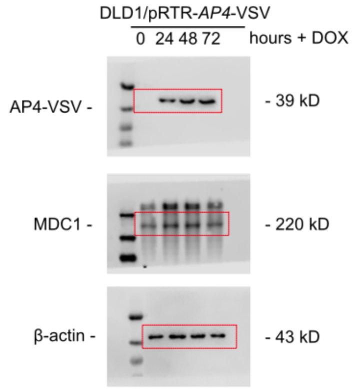

uncropped gel of Fig 4H and S4J

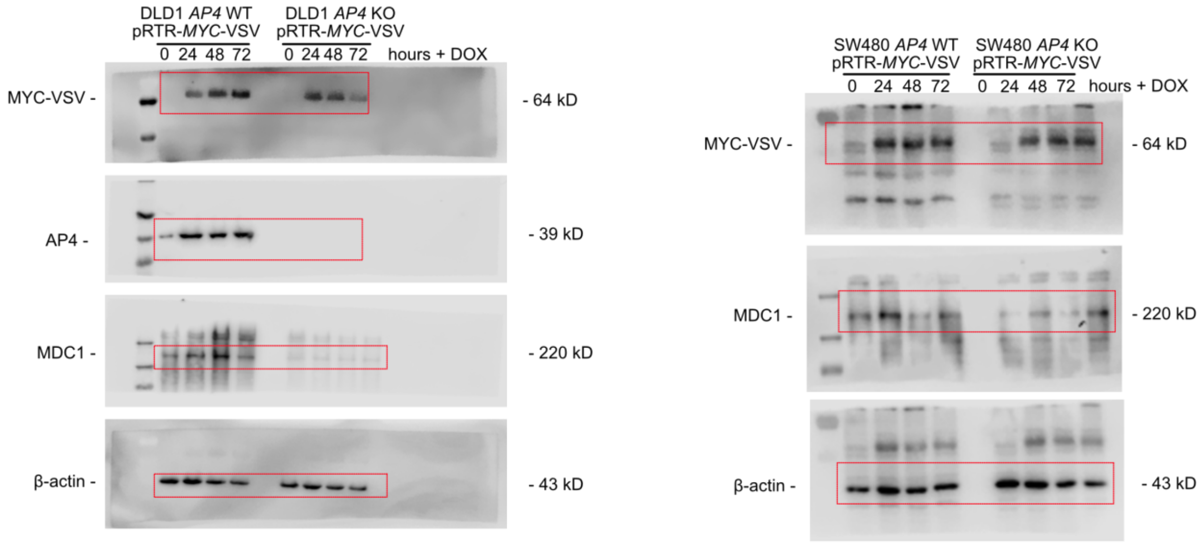

uncropped gel of Fig 9F

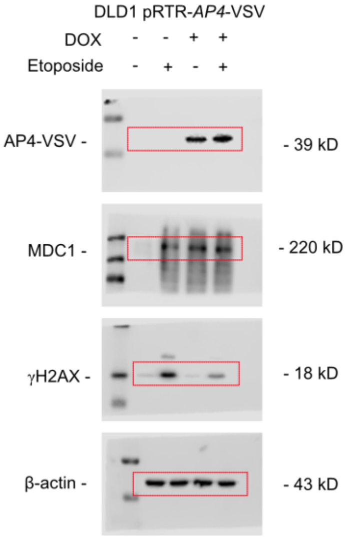

Uncropped gel for Figure S3C

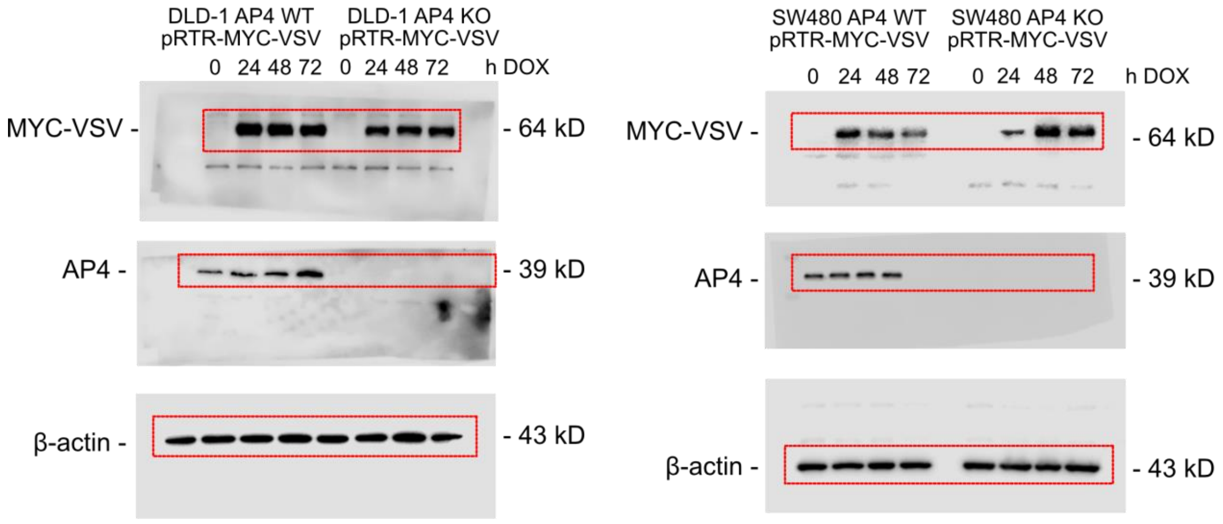

Table S1: Sequence information for miR-22-3p mimic and control mimic.

	Sequence information (5'-3')
hsa-miR-22-3p mimic	AAGCUGCCAGUUGAAGAACUGU
Control mimic	UCACCGGGUGUAAAUCAGCUUG

Table S2: Sequence information for guide RNAs used for *AP4* deletion.

	Sequence information (5'-3')
Guide RNA 1 Forward	CACCGACCAGGAGCGGCGGATTCGG
Guide RNA 1 Reverse	AAACCCGAATCCGCCGCTCCTGGTC
Guide RNA 2 Forward	CACCGGCGTCTCCGCTCGTTGCTGT
Guide RNA 2 Reverse	AAACACAGCAACGAGCGGAGACGCC
Guide RNA 3 Forward	CACCGCGCATGCAGAGCATCAACGC
Guide RNA 3 Reverse	AAACGCGTTGATGCTCTGCATGCGC

Table S3: List of Antibodies.

Epitope	Species	Catalog No.	Company	Use	Dilution	Source
Primary antibodies						
α-tubulin	Human	# T-9026	Sigma-Aldrich	WB	1:1000	mouse
β-actin	Human	# A2066	Sigma-Aldrich	WB	1:1000	rabbit
VSV	Human	# V4888	Sigma-Aldrich	WB	1:7500	rabbit
TFAP4	Human	# MCA4993Z	AbD Serotec	WB; ChIP	1:1000; 3 µg	mouse
MDC-1	Human	# NB100-397	NOVUS	WB/IF	1:1000	rabbit
Mdc-1	Mouse	# ab217528	Abcam	IHC	1:400	rabbit
γ-H2AX	Human	# JBW301	Sigma-Aldrich	IF	1:1000	mouse
γ-H2ax	Mouse	# 9718	Cell Signaling Technology	IHC	1:500	rabbit
p16 (M-156)	Mouse	# sc-1207	Santa Cruz	IHC	1:100	rabbit
Ki67	Mouse	# E0468	Dako	IHC	1:500	rat
F-actin	N.A.	# A12379	Thermo Fisher	IF	1:50	Alexa Fluor® 488 conjugated
Secondary antibodies						
Anti-mouse HRP	N.A.	# W4021	Promega	WB	1:10,000	goat
Anti-rabbit HRP	N.A.	# A0545	Sigma-Aldrich	WB	1:10,000	goat
Alexa Fluor Plus 555	N.A.	# A32727	Thermo Fisher	IF	1:1000	goat
Alexa Fluor Plus 488	N.A.	# A32731	Thermo Fisher	IF	1:1000	goat

Table S4: Oligonucleotides used for qPCR.

mRNA	Forward (5'-3')	Reverse (5'-3')
GAPDH	TGTTGCCATCAATGACCCCTT	CTCCACGACGTACTCAGCG
MDC1	TGCTCTTCACAGGAGTGGTG	GGGCACACAGGAACTTGACT
AP4	GCAGGCAATCCAGCACAT	GGAGGCGGTGTCAGAGGT
c-MYC	CTTCTCTCCGTCCTCGGATTCT	GAAGGTGATCCAGACTCTGACCTT
β-actin	TGACATTAAGGAGAAGCTGTGCTAC	GAGTTGAAGGTAGTTTCGTGGATG
MIR22HG	TTTGCAATAGGGGATTGCTT	TTTAATGTCTGCGCGGTACTC
p21	GGCGGCAGACCAGCATGACAGATT	GCAGGGGGCGGCCAGGGTAT
SNAI1	GCACATCCGAAGCCACAC	GGAGAAGGTCCGAGCACAC
mmu-*Ap4*	TCAAGCGCTTTATCCAGGAG	CAATGCCCTCATCCTTGTCT
mmu-*Mdc1*	CCACAAGAGCCAGGACCTTC	TGTAGCCAAGACTTCCCAAGG

Table S5: Oligonucleotides used for qChIP.

Gene	Forward (5'-3')	Reverse (5'-3')
MIR22HG (site A)	AGGGGGAGCAAATCACTGCG	CCGTGCATTCGCAGCTCGTG
MIR22HG (site B)	TGATGAGGCTGGAGGGTG	GGAGGGTAAGCAAGGAGGA
MIR22HG (site C)	AGGTCGGAGGTTGAGGAA	GTTGAGGCAGGCTGGAAG
MDC1 (site A and B)	GACAACCCACTACCGCTTGC	AAAGGCGCTCTGGCCTTACC
MDC1 (site C)	GAGATGACTTGTGGAATAGGAGGTAG	CCTTCCGGGACCTACCTCAG
SNAI1	GGAGTACTTAAGGGAGTTGGCGG	GAACCACTCGCTAGGCCGT
16q22	CTACTCACTTATCCATCCAGGCTAC	ATTTCACACACTCAGACATCACAG

Table S6: Oligonucleotides used for reporter plasmids.

Oligo	Forward (5'-3')	Reverse (5'-3')
MDC1 3'-UTR	AAACTAGTGAACTCCACTACCCTTTTC CCTC	GGCTGCAGTAAGGCACAGAGTGA ATATTTATTTATCA
miR-22-3p ΔSMS	TCATGCTCAGATGTCATAAGATCTTTA GCCAGACTGTTGC	GCAACAGTCTGGCTAAAGATCTTA TGACATCTGAGCATGA
miR-22-3p antisense	AATTCACAGTTCTTCAACTGGCAGCTT CTGCA	GTGTCAAGAAGTTGACCGTCGAA G
